# Clinical molecular subtyping reveals intrinsic mesenchymal reprogramming in gastric cancer cells

**DOI:** 10.1038/s12276-023-00989-z

**Published:** 2023-05-01

**Authors:** Eunji Jang, Min-Kyue Shin, Hyunki Kim, Joo Yeon Lim, Jae Eun Lee, Jungmin Park, Jungeun Kim, Hyeseon Kim, Youngmin Shin, Hye-Young Son, Yoon Young Choi, Woo Jin Hyung, Sung Hoon Noh, Jin-Suck Suh, Ji-Yong Sung, Yong-Min Huh, Jae-Ho Cheong

**Affiliations:** 1 MediBio-Informatics Research Center, Novomics Co., Ltd., Seoul, Republic of Korea; 2 College of Medicine, Yonsei University, Seoul, Republic of Korea; 3 Samsung Advanced Institute of Health Science and Technology, Sungkyunkwan University, Seoul, Republic of Korea; 4 Department of Pathology, Yonsei University, Seoul, Republic of Korea; 5 Department of Surgery, Yonsei University, Seoul, Republic of Korea; 6 Department of Radiology, Yonsei University, Seoul, Republic of Korea; 7 Department of Laboratory Medicine, Yonsei University College of Medicine, Seoul, Korea; 8 Department of Biomedical Systems Informatics, Yonsei University, Seoul, Republic of Korea; 9 YUHS-KRIBB Medical Convergence Research Institute, Seoul, Republic of Korea; 10 Department of Biochemistry & Molecular Biology, College of Medicine, Yonsei University, Seoul, Republic of Korea; 11 Brain Korea 21 Project for Medical Science, Yonsei University College of Medicine, Seoul, Republic of Korea

**Keywords:** Gastric cancer, Cancer genomics, Tumour heterogeneity, Cancer microenvironment, Classification and taxonomy

## Abstract

The mesenchymal cancer phenotype is known to be clinically related to treatment resistance and a poor prognosis. We identified gene signature-based molecular subtypes of gastric cancer (GC, *n* = 547) based on transcriptome data and validated their prognostic and predictive utility in multiple external cohorts. We subsequently examined their associations with tumor microenvironment (TME) features by employing cellular deconvolution methods and sequencing isolated GC populations. We further performed spatial transcriptomics analysis and immunohistochemistry, demonstrating the presence of GC cells in a partial epithelial-mesenchymal transition state. We performed network and pharmacogenomic database analyses to identify TGF-β signaling as a driver pathway and, thus, a therapeutic target. We further validated its expression in tumor cells in preclinical models and a single-cell dataset. Finally, we demonstrated that inhibition of TGF-β signaling negated mesenchymal/stem-like behavior and therapy resistance in GC cell lines and mouse xenograft models. In summary, we show that the mesenchymal GC phenotype could be driven by epithelial cancer cell-intrinsic TGF-β signaling and propose therapeutic strategies based on targeting the tumor-intrinsic mesenchymal reprogramming of medically intractable GC.

## Gastric cancer: Growth factor target for intractable cancer subtypes

Targeting a growth factor protein could reprogram tumor cells in difficult-to-treat gastric cancer, improving chemotherapy responses and prognosis. Several subtypes of gastric cancer are intractable and chemo-resistant. These often display elevated levels of genes expressed in mesenchymal stem cells, multipotent cells that are frequently involved in cancer progression. Jae-Ho Cheong and Yong-Min Huh at Yonsei University, Seoul, South Korea, and co-workers used gene expression profiling on samples from 547 patients to clarify gastric cancer molecular subtypes and their associated gene signatures. They validated their results against multiple external patient groups, and examined the association betweenf different subtypes and the tumour microenvironment. Experiments on mouse models showed that the transforming growth factor-beta pathway (TGF-β) drives the switch from normal to mesenchymal stem cell state of cancer cells not stormal cells. Inhibiting TGF-β signaling in gastric cancer cells blocked this transition and reduced chemotherapy resistance.

Introduction

The current standard of care for localized gastric cancer (GC) includes curative surgery followed by adjuvant chemotherapy to prevent disease recurrence and improve survival^1^. However, approximately one-third of cases treated with standard treatment reoccur within the 5-year follow-up^2^, indicating inconsistent therapeutic benefits. Biological tumor heterogeneity in GC contributes to the differences in the clinical course and outcomes of the disease. While microsatellite instability (MSI) and Epstein–Barr virus (EBV) positivity are associated with a favorable prognosis, genomically stable (GS) tumors demonstrate a poor prognosis and resistance to chemotherapy^3,4^. GS tumors are characterized by enrichment of diffuse histology, mesenchymal gene expression, and stem cell-like properties^5–7^. On the other hand, MSI is related to intestinal histology, epithelial gene expression, and proliferative features. In addition, EBV positivity and high MSI were predictive of an objective response to immune checkpoint inhibitors in a prospective clinical trial^8^. Although the chromosomal instability (CIN) subtype is generally considered to be responsive to chemotherapy and targeted therapies given its molecular characteristics of oncogene amplification, the CIN subtype is heterogeneous and has an unfavorable prognosis. These findings indicate that although there are unmet clinical needs for multiple TCGA subtypes, the GS and CIN subtypes should be prioritized in research considering their dismal prognosis and treatment resistance.

The tumor microenvironment (TME) composition is the key factor contributing to tumor heterogeneity. The stromal component and the activation of transforming growth factor (TGF)-β pathway in stromal cells have been associated with a poor prognosis in multiple cancer types, including GC and colorectal cancer (CRC)^9,10^. In particular, it has been demonstrated that cancer-associated fibroblasts (CAFs) promote the mesenchymal phenotypes of tumor cells via the TGF-β pathway^11,12^, implying that the composition of the stromal component alone could be clinically meaningful for classifying consensus molecular subtype (CMS) 4 in CRC. Moreover, epithelial-mesenchymal transition (EMT), which is critically regulated by TGF-β signaling, has been suggested as the key biological mechanism that is activated in tumor cells to promote invasion and metastasis^13^. Although some previous studies indicated that CRC may be resilient to TGF-β-induced EMT and that mesenchymal genes are mainly expressed by stromal cells but not by cancer cells^11,14^, another study reported activation of the TGF-β pathway in a cancer-cell intrinsic CRC subtype^15^. More recent studies using single-cell analysis demonstrated continuous regulation of EMT genes^16^ and revealed tumor cells within the intermediate EMT stages in head and neck squamous cell carcinoma and pancreatic ductal adenocarcinoma^17,18^. These results raise questions regarding the presence of a tumor cell-autonomous EMT state and its association with TGF-β signaling in GC.

We previously identified clinically relevant molecular subtypes and associated molecular signatures of GC to develop a clinical test for predicting the chemotherapy response of resectable GC^19^. In this study, we performed reanalysis of molecular subtypes, spatial transcriptome data, and RNA sequencing data of FACS-sorted GC cells to clarify the clinical implication of the TME, focusing on identifying the tumor cell-intrinsic mesenchymal phenotype. We discovered substantial differences in TME composition in the two subtypes with a dismal prognosis [Intestinal with stemness (INT/S) and Mesenchymal (MSC)] among 5 biological subtypes [Inflammatory (INF), Intestinal (INT), Gastric (GST), INT/S and MSC], but the EMT signature was expressed in both the INT/S subtype and the MSC subtype, indicating the presence of different molecular mechanisms driving the EMT phenotype in GC. Furthermore, spatial transcriptomics analysis supported the presence of cancer cells that have undergone EMT. Then, unbiased network analysis identified the TGF-β pathway as the therapeutic target to reverse mesenchymal gene expression in both subtypes. Finally, we tested a TGF-β inhibitor in medically refractory preclinical models, including patient-derived xenograft (PDX) models.

## Materials and methods

### Patient cohorts and datasets

We obtained fresh-frozen tumor specimens and clinical data from patients with GC who underwent gastrectomy as primary treatment at Yonsei Cancer Center (YCC, Seoul, South Korea) between 2000 and 2010. Using these samples, we then generated three cohort datasets (*n* = 547; GSE13861 [Illumina HumanWG-6 v3.0 expression beadchip], GSE84437 and GSE147163 [Illumina HumanHT-12 v3.0 Expression BeadChip array]). Published datasets, such as GSE15459, GSE62254 (ACRG), and TCGA (STAD), were downloaded from the Gene Expression Omnibus (GEO, https://www.ncbi.nlm.nih.gov/geo/) and Genomic Data Commons Data Portal (GDC, https://portal.gdc.cancer.gov/).

For analysis of the response to standard adjuvant chemotherapy, we pooled cohorts from the GSE13861, GSE26942, and GSE147163 datasets. Of the 178 patients with American Joint Committee on Cancer (AJCC) stage II or III GC, 121 patients received standard adjuvant chemotherapy (either single-agent 5-FU or a combination of 5-FU/capecitabine and cisplatin/oxaliplatin, doxorubicin, or paclitaxel). For analysis of the response to adjuvant chemotherapy or chemoradiotherapy, AJCC stage II and III patients were selected from the GSE62254 cohort. For comparative analysis of primary tumors and preclinical models, we obtained matched transcriptomic data from GSE98708 and GSE128459. For single-cell analysis, we took utilized the Tumor Immune Single-cell Hub^20^, a web resource that employs Single-Cell Signature Explorer for visualization of gene signatures^21^.

Transcriptomics and tumor growth curves of PDX models were obtained from HuBase (Crown Bioscience, CA, USA), an online PDX database. A total of 21 GC PDX models were treated with 5-FU (10 mg/kg, i.p., Day 1–5/week for 3 weeks) or vehicle. The response was considered significant if there was no overlap on the error bars on the last day.

### Subtype and module identification and characterization

Analyses were primarily conducted in the R language environment (RGUI version 3.5.3) or using web tools. Datasets were merged using the “Combating Batch Effects When Combining Batches of Gene Expression Microarray Data (ComBat)” method. Unsupervised clustering of patients and genes was conducted using the R package “Algorithms and Framework for Nonnegative Matrix Factorization (NMF)” and “WGCNA,”, respectively. For subtype clustering, the ComBat-merged data were classified using the package “Algorithms and Framework for Nonnegative Matrix Factorization (NMF)”. The number of clusters, k, was set from 3 to 7. The Brunet method was used as an updating algorithm for iterative approximation, and factorization was repeated 100 times for each condition. Prior to the characterization of NMF-derived clusters, we excluded outlier samples from each cluster using the Silhouette function in the “NMF” R package. To define genes significantly representative of each of the NMF clusters, SAM and PAM were conducted using the Bioconductor packages “siggenes” and “pamr”, respectively. For module annotation, WGCNA was conducted on the ComBat-merged data from 547 GC samples using the R “wgcna” package. For network construction, weighted network adjacency was defined by coexpression similarity with a power of 6. To avoid choosing an arbitrary cutoff, we followed the “soft-thresholding procedure” provided by WGCNA. Dynamic hybridization in the R “dynamicTreeCut” package was employed as a module detection method. To assess whether each module was associated with survival and clinicopathological variables, various module characteristics, such as connectivity, module significance, and module eigengene, were assessed. For annotation of subtypes and modules, gene set enrichment analysis was performed using the R “GSEABase” package, gene sets from the Molecular Signatures Database (MSigDB; www.broadinstitute.org/msigdb), and gene ontology (GO) information from the GO Consortium (http://geneontology.org). Single-sample GSEA was performed with GenePattern (https://www.genepattern.org/). Survival analysis was conducted using the Cox proportional hazard model and multivariate analysis in the R “survival” and “survivalAnalysis” packages, respectively.

### Immunohistochemistry

Paraffin-embedded tissue blocks were sliced into 4 μm sections and subjected to staining using a Ventana XT automated stainer (Ventana Corporation, Tucson, AZ, USA) with an anti-SFRP4 antibody (ab122905; Abcam, Cambridge, UK) as previously described^22^.

### Tumor microenvironment analyses

The estimated tumor purity, leukocyte fraction, and non-leukocyte stromal fraction of the STAD cohort samples were determined based on previously published data^23^. Briefly, sample purity was inferred from copy number variations (CNVs) using the ABSOLUTE^24^ algorithm to the whole-exome DNA sequencing data. The leukocyte fraction was extracted from DNA methylation data using methylation signatures of pure leukocyte cells^25^. Epithelial, immune, and fibroblast cell contents of the YCC cohort samples were measured with the xCell algorithm, which uses gene signatures derived from pure human cell type transcriptomes^26^. We performed RNA-seq of FACS-isolated cell populations from human primary GC samples (*n* = 9). Details are described in the supplementary information.

### Spatial transcriptomics (ST) analysis

ST sequencing of four GC primary tumors was performed with the Visium Spatial Gene Expression assay (10x Genomics, CA, USA) by its certified service provider (Geninus, Seoul, Republic of Korea). Fresh tissue was embedded in Optimal Cutting Temperature TissueTek (VWR, PA, USA) and stored at −80 °C until use. Tissues were tested using nProfiler 1 Stomach Cancer Assay (Novomics, Seoul, Republic of Koera) to classify into the Single Patient Classifier subtype^19^. Using Space Ranger software v1.1.0 and Loupe Browser software v5.0, we performed analysis of a sample that included normal epithelium, the cancer region, stroma, and lymphoid tissue.

### Network analysis

Ingenuity Pathways Analysis (Ingenuity Systems, www.ingenuity.com) was used to identify the upstream transcriptional regulators that can explain the gene expression differences between GC subtypes. The expression log ratio of PAM genes in the YCC cohort was used for analysis. The Connectivity Map (https://clue.io/) was queried to identify reference perturbagen signatures most similar (positive score) or dissimilar (negative score) to each GC module.

### Cell sorting from GC specimens and RNA sequencing

Primary tumor tissues were minced, and single-cell suspensions were obtained. A detailed procedure is provided in the supplementary methods. The collected cells were incubated with antibody for 30 min on ice as follows: 10 μL anti-EpCAM (R&D Systems, FAB9601F) per 106 cells in 100 μL buffer; 5 μL anti-CD45 (BD, 557748) per 106 cells in 100 μL buffer; 10 μL anti-CD31 (Miltenyi Biotec, 130-092-652) per 107 cells in 100 μL buffer; and 2.5 μg of FAP (R&D systems, MAB3715) per 106 cells in 100 μL buffer. After washing with Hank’s balanced salt solution (Lonza) twice, the cells were incubated with mouse IgG (H + L) PE as follows: 10 μL per 106 cells in 100 μL buffer for 30 min on ice. After washing away the unstained secondary antibody, the cells were resuspended in 1 mL PBS and then sorted using a BD FACSARIA III (BD Biosciences). Total RNA sequencing libraries were prepared according to the manufacturer’s instructions (Illumina TruSeq RNA Access Library kit). The flow cell was then loaded on a HiSeq 2500 sequencing system (Illumina), and sequencing was performed using 2 × 100 bp read lengths.

### In vitro and in vivo experimental validation

We generated gene expression profiles of 27 GC cell lines (GSE146361; Illumina HumanHT-12 v3.0 Expression BeadChip array). Details are described in the supplementary information. For experimental validation, invasion assays, migration assays, tumor spheroid assays, and in vivo orthotopic tumorigenicity assays were performed using GC cell lines.

### Invasion assay

For this assay, 2 × 10^4^ HUVECs in culture medium (M199) were added to the upper chamber of a transwell plate coated with fibronectin (the bottom chamber was coated with 0.2% gelatin) and subsequently incubated for 48 h until the formation of a monolayer. Thereafter, 1 × 10^5^/50 µL Hs746T and NCI-N87 cells incubated with CellTracker™ (Molecular Probes, C2925) without FBS were separately added to the upper transwell chamber. Culture medium with 10% FBS was added to the lower chamber. After incubation for 48 h, the cells on the upper membrane were removed with a cotton swab, and the cells on the lower membrane were lysed with 200 µL of lysis buffer for 2–3 h at room temperature. Fluorescence was measured with an Ex/Em of 492/517. To examine the effect of the TGF-beta inhibitor on the invasion ability of cells, 50 µM LY2157299 (AdooQ, CA, USA) was administered.

### Migration assay

Hs746T and NCI-N87 cells were grown into monolayers in culture media with 10% FBS and 1% antibiotics. When the confluency reached 70%, the cell monolayers were scratched with a 100 μL pipette tip. Wound width was measured after 72 h and then normalized to wound width measured immediately after scratching. To assess the effect of the TGF-β inhibitor on cellular migration, LY2157299 (50 µM) was administered.

### Tumor spheroid assay

In 96-well plates, 10 cells were cultured in 50 μL DMEM/F12 (Gibco) supplemented with bFGF, EGF, B27, 10% FBS, and 1% antibiotics. After 30 days of incubation, spheres were counted in each well. Additionally, LY2157299 (50 µM) was administered to investigate the effect of a TGF-β inhibitor on tumor spheroid formation.

### In vivo tumorigenesis in an orthotopic mouse model

To establish the orthotopic xenograft mouse model, 1 × 10^7^ GC cells (Hs746T and NCI-N87) were transplanted into the walls of the stomachs of BALB/c nude mice (male, 6 weeks old, 20–24 g) exteriorized by incision of the skin and peritoneum along the upper midline for approximately 5 mm. The stomach was returned to the peritoneum, and the abdominal wall was closed with a wound suture in one layer. To observe tumor growth in the model, we performed in vivo magnetic resonance imaging (MRI) experiments using a 9.4 T animal MRI instrument with a Bruker animal coil (RF SUC 400 1H M-BR-LIN ROAD, Bruker Medical Systems). The sequences were performed at room temperature with the following parameters: Echo = 1, TR = 2300 ms, TE = 22.0 ms, FA = 180 deg, TA = Oh4m54s400ms, NEX = 2, and FOV = 4.00 cm.

### Drug response in xenograft mouse models

To establish the heterotopic xenograft mouse model, 1 × 10^7^ GC cells (Hs746T and NCI-N87) were transplanted into the proximal thigh region of BALB/c nude mice (male, 6 weeks old, 20–24 g). Tumor-bearing mice were randomly assigned to three groups for treatments (PBS control, Oxal + 5FU/PBS, and Oxal + 5FU/LY-treated groups; *n* = 8 per group) when the tumor volume increased to approximately 400 mm^3^. Oxaliplatin (60 µg per single dose) and fluorouracil (1 mg per single dose) in combination were intraperitoneally injected into mice three times per week. LY2157299 (1.5 mg per mouse) was administered to the mice by intratumor injection two times per week. All administrations were blinded to group assignment and outcome assessment. The size of the implanted tumor was assessed three times per week and calculated as follows: (4/3) × π × (minor axis/2)^2^ × (major axis/2) mm^3^. Protocols for establishing the PDX models were previously described^27^.

### Statistical analysis and visualization

We performed the hypergeometric test/Fisher’s exact test, Pearson’s correlation, point-biserial correlation, Spearman’s correlation, Student’s t test, and the Wilcoxon rank-sum test for statistical analysis using R. The center values and error bars represent the mean and standard deviation, respectively. We used 5% as the significance level for all tests. For visualization, the R packages “ggplot2” and “ggpubr” were used.

## Results

### Clinically relevant molecular subtypes of gastric cancer

**Fig. 1:**
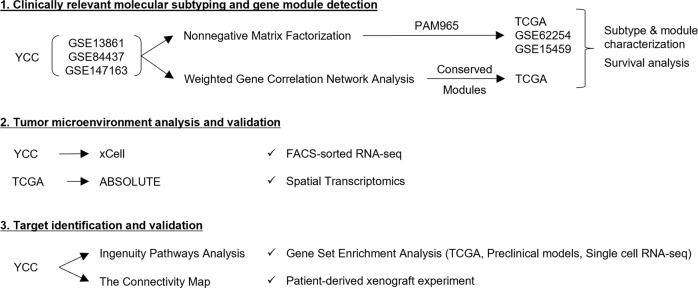
Study Profie. Flow chart of this study.

**Fig. 2:**
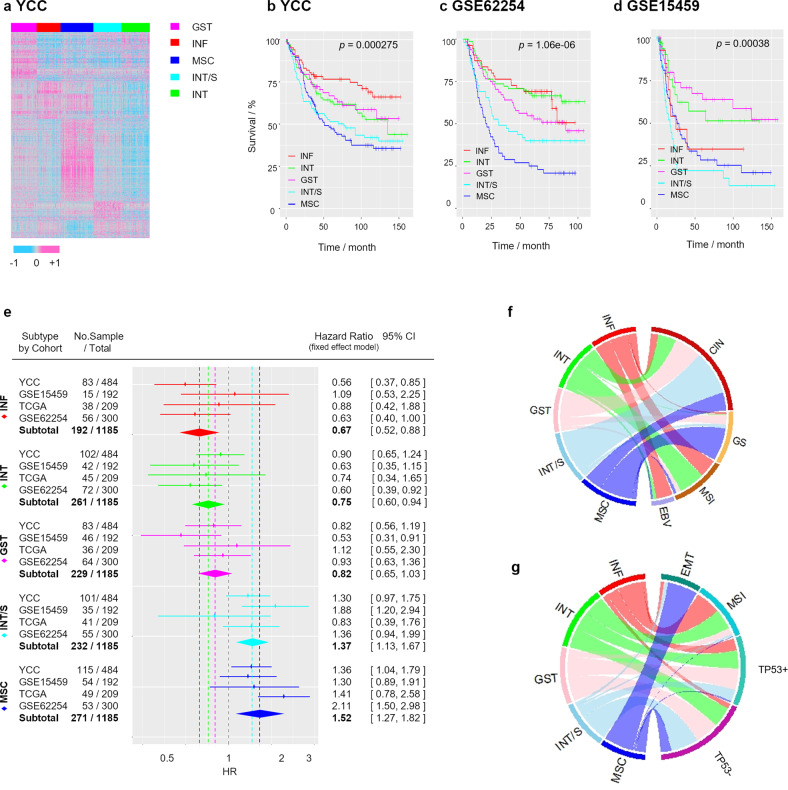
Molecular subtyping stratifies gastric cancer (GC) patients based on clinical prognosis. **a** Nonnegative matrix factorization (NMF) consensus clustering of the YCC dataset (*n* = 547). **b**–**d** Overall survival curves of patients in the **b** YCC, **c** GSE62254, and **d** GSE15459 cohorts stratified by NMF consensus clustering using Classifier-PAM965. **e** Forest plot generated by meta-analysis of the hazard ratio (HR) for overall survival (OS) according to the five subtypes using a fixed-effects model (*P* > 0.05 for all subtypes in the heterogeneity test). **f**, **g** Chord diagram for the NMF subtype in **f** the TCGA cohort and **g** GSE62254 cohort (EBV Epstein–Barr virus, MSI microsatellite instability, GS genomically stable, CIN chromosomal instability, EMT epithelial-mesenchymal transition).

A study flow chart is shown in Fig. 1. We obtained fresh-frozen tumor specimens and clinical data from GC patients who underwent gastrectomy as a primary treatment at the Yonsei Cancer Center (YCC, Seoul, South Korea). Using the YCC cohort dataset (*n* = 547; GSE13861, GSE84437, and GSE147163), we clustered the patients and genes using the consensus-based NMF method^28^. The NMF method classified GC patients into five distinctive molecular subtypes with high consensus (ρ5 > 0.99) (Fig. 2a and Supplementary Fig. 1; prediction analysis of microarray (PAM) genes and their scores are listed in Supplementary Data 1; see Supplementary Results and Discussion for further information). Based on the expression of genes relevant to GC biology and the results from gene set enrichment analysis (GSEA; Supplementary Table 1), we named each subtype as follows: (i) gastric (GST): characterized by high expression of gastric-specific genes *TFF1, TFF2*, and *GKN1*; (ii) inflammatory (INF): characterized by high expression of immune genes, including *CXCL9, GBP5*, and *NKG7*; (iii) mesenchymal (MSC): characterized by high expression of myogenetic (*MYLK* and *MYH11*) and EMT (*SFRP1* and *TAGLN*) genes; (iv) intestinal (INT): characterized by high expression of cell cycle (*CDC20* and *AURKA*)- and intestinal epithelial differentiation (*CDH17* and *CDX1*)-related genes; and (v) intestinal with stem-like features (INT/S): characterized by high expression of cell cycle and intestinal epithelial differentiation-related genes, as well as EMT-related genes (*COL11A1* and *CTHRC1*) and Wnt signaling genes (*NKD2* and *DKK3*).

We examined the relationships between the GC subtypes and clinicopathological factors. The distributions of the five subtypes were significantly different in samples grouped based on the following categories (hypergeometric test; Supplementary Table 2): patient age (*P* = 0.0003), pathological T stage (*P* = 0.007), World Health Organization (WHO) classification (*P* = 1.4e-14), and Lauren type (*P* = 2.4e-11). Survival analysis identified distinct clinical outcomes between subtypes (likelihood ratio test; *P* = 0.000275; Fig. 2b). The 5-year survival rate was 76.7% for the INF subtype (95% CI, 67.8–86.4%), 63.1% for the INT subtype (95% CI, 54.1–73.6%), 66.8% for the GST subtype (95% CI, 56.9–78.4%), 51.4% for the INT/S subtype (95% CI, 42.4–62.4%), and 47.1% for the STL subtype (95% CI, 38.6–57.5%). We performed NMF analysis in independent cohorts (GSE15459 and GSE62254) using PAM genes derived from the YCC cohort, and this demonstrated consistently poor outcomes for the MSC and INT/S subtypes (Fig. 2c, d). A meta-analysis of the hazard ratio (HR) for all cohorts revealed that the NMF classification effect was homogenous (effect-equality test; *P* > 0.01) and that the HRs indicated significant differences in risk among the GC subtypes (Fig. 2e): INF, 0.68 (95% CI 0.52–0.89); INT, 0.75 (95% CI 0.60–0.94); GST, 0.82 (95% CI 0.65–1.03); INT/S, 1.37 (95% CI 1.13–1.67); and MSC, 1.52 (95% CI 1.27–1.82).

We compared the GC subtypes with other molecular subtypes reported in previous studies (Fig. 2f, g and Supplementary Table 3). Whereas the MSC subtype overlapped with a specific subtype in all other classification systems (the TCGA GS subtype^4^, the ACRG EMT subtype^7^, and the invasive/mesenchymal subtype reported by Lei et al.^5^), the INT/S subtype did not correspond with a specific subtype. Rather, it was a subset of the TCGA CIN subtype, ACRG MSS subtype, or the proliferative subtype^5^ and was not separated from the INT and GST subtypes.

### Association between molecular subtypes and the tumor microenvironment

**Fig. 3:**
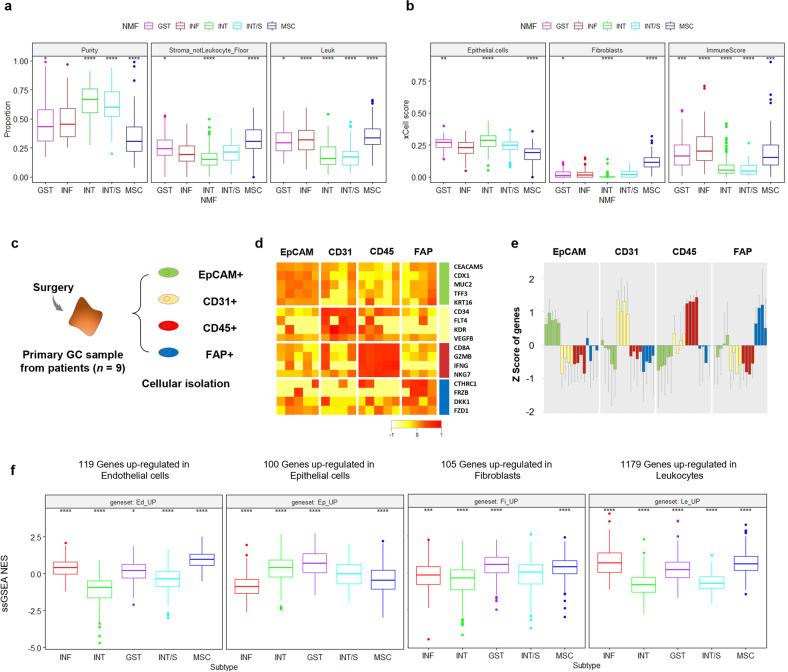
Association between molecular subtypes and the tumor microenvironment. **a** Tumor purity, nonleukocyte stromal fraction, and leukocyte fraction estimated by the ABSOLUTE algorithm in the STAD cohort (*n* = 399). **b** xCell scores of epithelial cells, fibroblasts, and immune cells in the YCC dataset. (*P ≤ 0.05, **P ≤ 0.01, ***P ≤ 0.001, ****P ≤ 0.0001, Wilcoxon test against all) (centerline, median; box limits, upper and lower quartiles; whiskers, 1.5× interquartile range; points, outliers). **c** The specific cell types were purified from disaggregated primary GC samples by FACS using the indicated markers: EpCAM for epithelial cells, CD31 for endothelial cells, CD45 for leukocytes, and FAP for fibroblasts. **d** The mean expression levels and **e** z scores of epithelial (CEACAM5, CDX1, MUC2, TFF3, and KRT16), endothelial (CD34, FLT4, KR, and VEGFB), leukocyte (CD8A, GZMB, IFNG, and NKG7), and fibroblast (CTHRC1, FRZB, DKK1, and FZD1) genes in each FACS-purified cell population are shown in the heatmap (TMM normalization). **f** Enrichment of cell type-specific gene expression in the merged cohort (YCC, TCGA, GSE62254, GSE15459, *n* = 1412) according to molecular subtype (single-sample GSEA, ssGSEA; normalized enrichment score, NES). Each gene set was identified by Student’s t test (*P* < 0.01).

We investigated the association between GC subtypes and TME composition. First, tumor purity was inferred from somatic DNA alterations with the ABSOLUTE algorithm, and the leukocyte fraction was measured using DNA methylation signatures in the TCGA cohort (Fig. 3a)^24,25^. Compared to other subtypes, the MSC subtype demonstrated significantly lower tumor cell purity while displaying a significantly higher fraction of both leukocyte and non-leukocyte stroma. In contrast, the INT/S subtype exhibited significantly higher tumor purity but a lower stromal fraction, similar to the INT subtype. Next, we applied the transcriptome-based xCell algorithm to the YCC cohort (Fig. 3b)^26^. Concordantly, the MSC subtype showed significantly lower epithelial cell scores but significantly higher fibroblast and immune cell scores. On the other hand, the INT/S subtype did not exhibit a significant difference in the fractions of epithelial cells or fibroblasts, but its immune score was significantly lower that than of other subtypes, at a level similar to that of the INT subtype. Finally, we performed RNA sequencing of cell populations isolated from GC tissue (*n* = 9) based on four markers (*EpCAM* for epithelial cancer cells, *CD45* for leukocytes, *CD31* for endothelial cells, and *FAP* for CAFs) (Fig. 3c). Each cell population showed differential expression of cell type-specific marker genes (Fig. 3d, e). Then, we identified genes upregulated in each cell type and examined their enrichment in the five molecular subtypes in the merged cohort (YCC, TCGA, GSE62254, GSE15459, *n* = 1412, Fig. 3f). When normal tissues and GC tissues were compared, the MSC subtype showed increased enrichment of genes upregulated in stromal cells (endothelial cells, fibroblasts, and leukocytes) but decreased enrichment of genes upregulated in epithelial cells. On the other hand, the INT subtype showed increased enrichment of genes upregulated in epithelial cells but decreased enrichment of genes upregulated in stromal cells. The INT/S subtype demonstrated low expression of genes upregulated in endothelial cells and leukocytes, while the INF subtype showed high expression of genes upregulated in endothelial cells and leukocytes.

### Discovery of molecular signatures describing GC subtypes

**Fig. 4:**
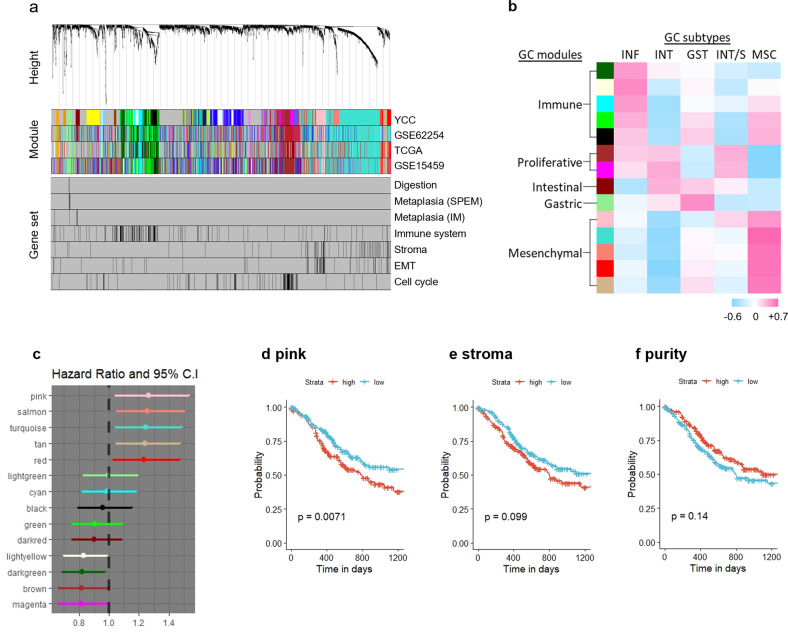
Subtype-defining gene modules and their prognostic association. **a** Dendrogram from the weighted correlation network analysis (WGCNA) of the YCC dataset. Modules detected in the YCC dataset and corresponding module maps of the GSE62254, TCGA, and GSE15459 datasets. Mapping of the predefined gene sets. **b** Heatmap of point-biserial correlation between the five GC subtypes and conserved GC signatures. GST, gastric; INF, inflammatory; MSC, mesenchymal; INT/S, intestinal with stem-like features; INT, intestinal. **c** Multivariate analysis (overall survival) of the normalized enrichment score (NES) for each GC module in the STAD cohort adjusted for age, sex, race (Asian vs. Non-Asian), AJCC stage, histologic grade, and histologic type (Diffuse vs. Non-Diffuse). **d**–**f** Overall survival of the STAD cohort stratified by **d** pink module NES, **e** nonleukocyte stromal fraction, and **f** tumor purity estimated by the ABSOLUTE algorithm. Median values were applied as thresholds.

Considering the distinct TME composition of the two dismal subtypes, we performed unsupervised genewise clustering to reveal de novo gene modules and assessed their association with molecular subtypes. Weighted correlation network analysis (WGCNA) detected 32 gene modules in the YCC cohort (Supplementary Data 2), and these gene modules were relevant to GC biology [digestion^29^, spasmolytic polypeptide-expressing (SPEM) and intestinal metaplasia (IM) lineages^30,31^, immune system^32^, extracellular matrix regulation^33^, angiogenesis^34^, EMT^35^, Wnt signaling pathway^36^, and the cell cycle^37^] (Fig. 4a). We selected 14 modules that were conserved across independent cohorts (hypergeometric test; *P* < 0.01) and categorized them into five core GC signatures based on module eigengene analyses (Supplementary Fig. 2) and gene ontology enrichment analysis (Supplementary Table 4): (i) the immune meta-module (modules depicted as black, green, cyan, light yellow, and dark green colors in the figure), which is highly associated with the immunoregulatory system; (ii) the mesenchymal meta-module (turquoise, red, tan, pink, and salmon), which is closely related to the stromal component, angiogenesis, EMT, TGF signaling, Wnt signaling, and the extracellular matrix regulatory system; (iii) the proliferative meta-module (brown and magenta modules), which is associated with the cell division regulatory system; (iv) the gastric module (light green module) which is indicated by gastric epithelial markers and associated with digestion; and (v) the intestinal module (dark red module), which is indicated by intestinal epithelial markers. In particular, the pink module among the mesenchymal meta-module predominantly overlapped with the predefined EMT gene set. A correlation analysis confirmed a significant association between EMT marker protein expression and the pink module enrichment score (pink score) calculated by single-sample GSEA (ssGSEA) of the TCGA dataset (Supplementary Fig. 3).

Then, we performed point-biserial correlation analysis between the conserved modules and the five subtypes, which identified remarkable associations between mesenchymal modules and the MSC subtype (Fig. 4b). Notably, only the pink module was positively correlated with both unfavorable subtypes—the MSC and INT/S. A significant association between poor prognosis and the mesenchymal meta-module enrichment score was confirmed with multivariate analysis adjusted for age, sex, race, AJCC stage, histologic grade, and histologic type in the TCGA cohort (Fig. 4c). Furthermore, we evaluated the associations of overall survival with the stromal score and tumor purity measured by the ABSOLUTE algorithm in the TCGA cohort. While a high pink score was significantly associated with short survival (*P* = 0.0071), the stromal score (*P* = 0.099) and tumor purity (*P* = 0.14) were not significantly associated with survival outcome (Fig. 4d–f).

### Association between molecular signatures and the tumor microenvironment

**Fig. 5:**
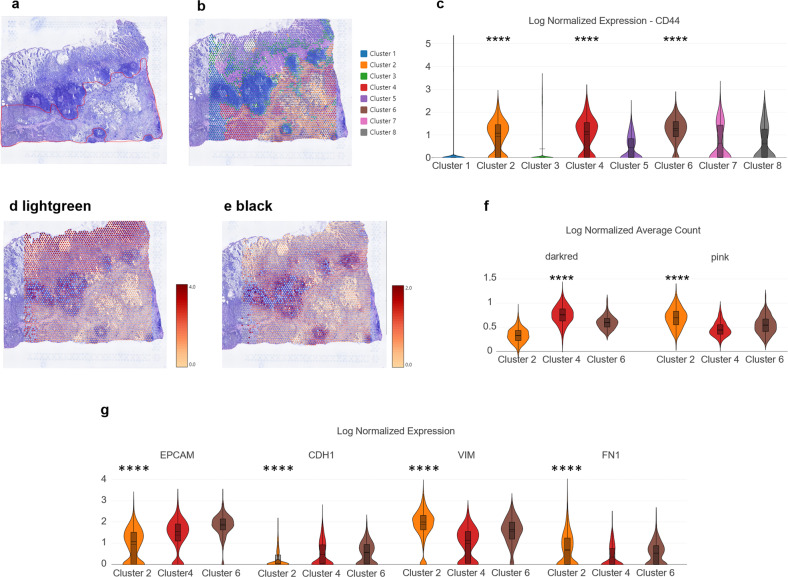
Implication of the pink module in EMT demonstrated by spatial transcriptomic analysis. **a** Pathological annotation of malignant lesions in a H&E slide of GC tissue. **b** Graph-based clustering of the Visium data. **c** Violin plot demonstrating the log-normalized expression of CD44, a cancer stem cell marker, for each cluster. **d**, **e** Log-normalized average count of gene modules displayed by color coding. Red represents high expression, whereas yellow represents low expression. **f** Violin plots representing the log-normalized average count of gene modules for Clusters 2, 4, and 6. **g** Violin plots for epithelial (EPCAM and CDH1) and mesenchymal (VIM and FN1) marker genes (****P ≤ 0.0001, Wilcoxon test for these genes vs. all genes).

We analyzed the spatial transcriptomics of a primary GC sample, which was classified as the high-risk type using Single Patient Classifier^19^, to study the associations between the tumor microenvironment and the molecular signatures that we discovered. First, an individual pathologist annotated the malignant portion in the hematoxylin and eosin (H&E)-stained slide (Fig. 5a and Supplementary Fig. 4). Then, tissue spots were clustered by gene expression similarity based on a nearest-neighbor graph approach (Fig. 5b). When we looked at the log-normalized expression of *CD44*, a well-recognized marker of cancer stem cells^38^ that is not expressed in benign mucosa^39^, it was highly expressed in Clusters 2, 4, and 6, which were pathologically annotated as malignant lesions (Fig. 5c). Partial expression of *CD44* in Clusters 5 and 7 can be explained by leukocyte infiltration observed in the H&E slide, which suggests chronic inflammation accompanied by cancer^39^. Next, we identified high expression of the gastric (light green) module in benign mucosa and the immune (black) module in lymphoid follicles (Fig. 5d, e). Among the malignant cell clusters, Cluster 4 highly expressed the intestinal (dark red) module, while Cluster 2 highly expressed the mesenchymal (pink) module (Fig. 5f). Concordantly, epithelial marker genes (EPCAM and CDH1) demonstrated higher expression in Cluster 4 than in Cluster 2, while mesenchymal marker genes (VIM and FN1) displayed higher expression in Cluster 2 than in Cluster 4 (Fig. 5g). Moreover, Cluster 6 moderately expressed both dark red and pink modules and showed coexpression of EPCAM and VIM. Additionally, protein expression analysis of a different GC sample revealed that epithelial cancer cells showed coexpression of cytokeratin with *SFRP4* (Supplementary Fig. 5), which belongs to the pink module and was top-ranked in a correlation analysis between the matched transcriptome and proteome dataset (GSE122401). Altogether, these data indicate that the intratumoral heterogeneity of GC might originate from the intermediate EMT status, which is represented in the GC modules derived from intertumoral analysis.

In addition, we examined the association of the pink module with stem-like characteristics in epithelial cells using external datasets. First, human gastric pyloric stem cells, isolated based on the expression of *AQP5*, showed higher expression of the pink module genes than *AQP5*- cells (Supplementary Fig. 6a)^40^. Second, multipotent endodermal progenitors induced from human gastric epithelial cells demonstrated upregulation of pink module genes (Supplementary Fig. 6b)^41^. Apart from GC, in transcriptomic datasets of breast cancer metastasis models, epithelial cells within the metastatic niche exhibited higher expression of the pink module genes than distal epithelial cells (Supplementary Fig. 7a), and the same pattern was observed for disseminated indolent cancer cells compared to proliferative cancer cells (Supplementary Fig. 7b). These results indicate that the pink module genes may be implicated in the stemness features of epithelial cells.

### The pink module is associated with the responsiveness to chemotherapy

Given that the pink module is associated with a poor prognosis and mesenchymal/stemness features, we further examined whether it is also associated with the response to adjuvant treatments. First, we classified the patients in the ACRG cohort, who received adjuvant chemotherapy ± radiotherapy or surgery alone (*n* = 193; stage II–III patients from GSE62254), based on the pink score (Supplementary Fig. 8a). We observed a significant benefit (*P* = 0.0028) from adjuvant treatments in patients with a low pink score, whereas no benefit (*P* = 0.3) was observed among patients with a high pink score. Moreover, when the ACRG cohort was stratified by stromal score calculated by xCell analysis^26^, both subgroups showed moderate benefit (*P* = 0.039 and *P* = 0.035) (Supplementary Fig. 8b). Additionally, when the patients were stratified by tumor purity measured based on SNP array data (GSE62717) with ASCAT analysis^42^, there was no statistically significant benefit (*P* = 0.11) from adjuvant treatments in patients with high tumor purity (Supplementary Fig. 8c). Next, we classified a pooled cohort treated with standard adjuvant chemotherapy or surgery alone (*n* = 178; stage II–III patients from GSE13861, GSE26942, and GSE147163) in the same manner. We similarly found that only patients with a low pink score showed a survival benefit (*P* = 0.011 vs. *P* = 0.55) from chemotherapy, and there were no significant differences when the patients were stratified by stromal score (*P* = 0.15 and *P* = 0.13) (Supplementary Fig. 8d, e). These findings reemphasize that the pink module gene score is more clinically significant than the stromal score or tumor purity.

Along the same lines, we examined a previously established pharmacogenomic database of PDX models, where fresh tumor fragments were directly implanted into BALBC/nude mice^43^. The 21 PDX model mice were treated with a fixed dose of a standard GC drug (5-FU) or control; we compared their responses to 5-FU and the vehicle and queried the expression levels of the pink module genes (Supplementary Data 3 and Supplementary Fig. 9a). Compared to the chemosensitive models, the PDX models refractory to 5-FU treatment demonstrated significant (*P* = 0.041) upregulation of pink module genes (Supplementary Fig. 9b). Additionally, in cohorts in which patients received adjuvant chemoradiotherapy or palliative chemotherapy, we witnessed a trend of poor prognosis among the patients with high pink scores (Supplementary Fig. 9c, d).

### Regulation of the pink module by TGF-β signaling in refractory subtypes

**Fig. 6:**
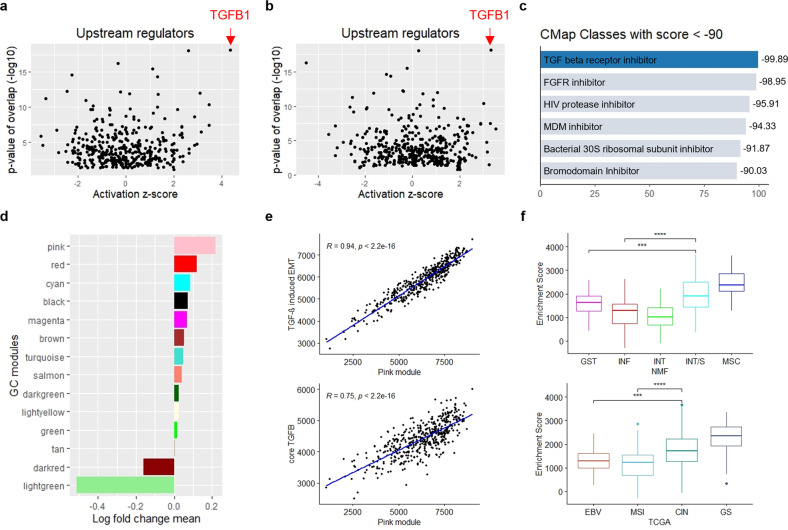
TGF-β signaling regulates pink module gene expression in refractory subtypes. **a**, **b** Top 5 activated upstream regulators identified by Ingenuity Pathway Analysis of **a** the MSC subtype and **b** the INT/S subtype. Expression log ratio of Classifier-PAM965 genes in the YCC cohort was used as input. **c** Compounds reversing the pink module gene expression identified by the Connectivity Map. **d** Log fold change mean of conserved GC module genes in TGF-β1-treated gastric epithelial cell lines compared with untreated cell lines (GSE44055; three replicates for each group). **e** Correlation analysis of the pink module enrichment score with the pancancer TGFβ-induced EMT signature and core TGF-β signaling genes in the TCGA cohort (*n* = 450). **f** Core TGF-β signaling enrichment scores according to NMF and TCGA subtypes in the TCGA cohort (***P ≤ 0.001, ****P ≤ 0.0001, Wilcoxon test) (centerline, median; box limits, upper and lower quartiles; whiskers, 1.5× interquartile range; points, outliers).

On the basis of the subtypes and modules studied above, we identified a potential therapeutic target among the poor prognostic subtypes via network analyses. First, Ingenuity Pathways Analysis, a knowledge-based bioinformatic analysis software^44^, revealed that *TGFB1* was the top upstream regulator of both the MSC and INT/S subtypes (Fig. 6a, b). Second, TGF-β receptor inhibitor was identified as the top opposing perturbagen against the pink module genes according to the Connectivity Map, a gene expression database of cancer cell lines treated with chemical and genetic perturbations^45^ (Fig. 6c). In addition, TGF-β receptor inhibitor was one of the top inducing perturbagens for epithelial and proliferative modules (enrichment scores of 95.7 for light green, 93.04 for dark red, 99.89 for brown, and 99.83 for magenta), supporting its actionability against EMT and dormancy. Indeed, the “cellular response to TGF-β stimulus” was one of the significantly enriched biological processes of the pink module (Supplementary Table 4). Accordingly, we observed that the growth-inhibitory activities of three TGF-β receptor inhibitors were significantly (*P* < 0.05) associated with the pink score in human cancer cell lines from the PRISM Repurposing dataset^46^ (Supplementary Table 5). Furthermore, the pink module was the most upregulated GC module in the TGFβ-induced EMT model of a gastric epithelial cell (GSE44055)^47^, whereas both epithelial (dark red and light green) modules were the most downregulated GC modules (Fig. 6d). Finally, the pink score was highly correlated with the enrichment scores of a TGFβ-induced EMT signature (R = 0.94), which were derived from a meta-analysis of pancancer datasets^48^, and the levels of the core TGF-β signaling genes (R = 0.75), which were selected by consensus of TCGA network members^49^ (Fig. 6e). These findings suggest that TGF-β signaling regulates the pink module genes, which is also in accordance with their implications in EMT status.

We also examined whether the TGF signaling gene set itself is associated with poor-prognosis-related GC subtypes and whether it has prognostic value. The core TGF-β signaling gene set enrichment score (core TGF-β score) was highest in the MSC subtype and then the INT/S subtype among the NMF subtypes that we identified, and it was highest in the GS subtype and then the CIN subtype among the TCGA subtypes (Fig. 6f). In addition, the core TGF-β score was significantly (*P* = 0.011) associated with a shorter disease-free interval in TCGA CIN subtype patients (*n* = 122) (Supplementary Fig. 10a). Moreover, among the MSS/epithelial subtype patients in the ACRG cohort (*n* = 123), patients with high core TGF-β scores were resistant to adjuvant therapy, while patients with low core TGF-β scores were sensitive to adjuvant therapy (Supplementary Fig. 10b). Together, these findings indicate that TGF-β signaling gene expression could separate patients with poor clinical outcome from a population of nonmesenchymal tumors.

### TGF-β inhibition against mesenchymal phenotypes and chemoresistance in preclinical models

**Fig. 7:**
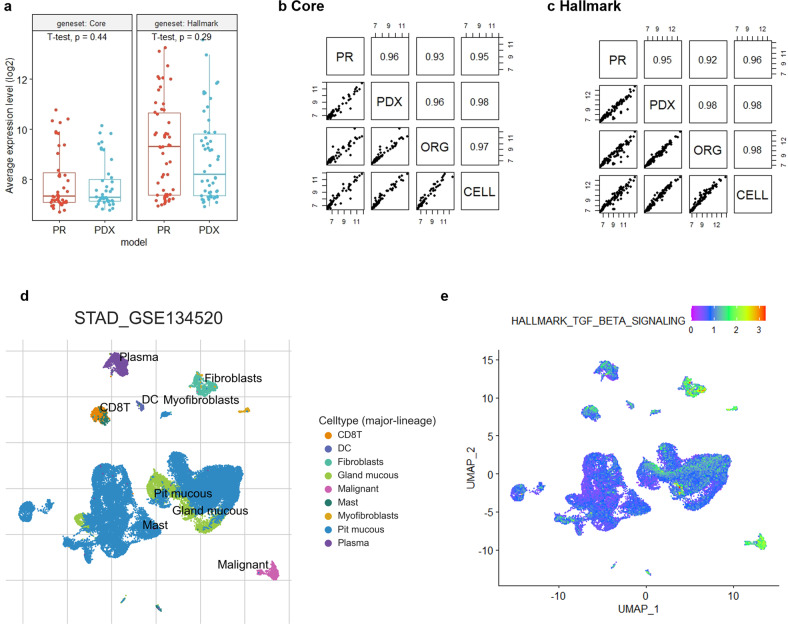
TGF-β signaling factors are intrinsically expressed by GC cells. **a** Box plot comparing, for core and hallmark TGF-β signaling genes tested on human arrays, average expression in human primary (PR) GC samples and corresponding PDX derivatives (*n* = 18) (GSE98708). **b**, **c** Correlation plot matrix comparing, for **b** core and **c** hallmark TGF-β signaling genes tested on human arrays, average expression in matched GC models (*n* = 7) (GSE128459). ORG, organoids derived from xenografts; CELL, frozen cells derived from xenografts. **d** UMAP plot of GSE134520 with major-lineage cell-type annotation from Tumor Immune Single-cell Hub (TISCH). **e** Gene set enrichment analysis (GSEA) score of hallmark TGF-β signaling from Single-Cell Signature Explorer.

We investigated whether TGF-β signaling pathway is intrinsically induced by cancer cells using transcriptomic data from matched primary tumors and preclinical models. Since stromal cells in the xenografted TME are substituted for mouse cells, it is assumed that human microarray profiling of PDX samples does not detect transcripts of stromal origin^10^. Interestingly, the expression levels of both the core TGF-β signaling genes and the hallmark TGF-β signaling gene set^50^ were conserved upon tumor transplantation in GC PDX models (GSE98708^51^) (Fig. 7a). Moreover, the expression levels of both TGF-β gene sets were highly (*R* > 0.9) correlated not only between the primary tumor and the PDX but also between organoids and frozen cells derived from the PDX (GSE128459^51^) (Fig. 7b, c), and the two latter models do not include stromal cells. Finally, we interrogated single-cell RNA sequencing (scRNA-seq) data for GC (GSE134520) with cell type annotation based on gene signatures^20^ (Fig. 7d). Notably, GSEA demonstrated that malignant cells are enriched with the hallmark TGF-β signaling pathway as much as CAFs (Fig. 7e). Collectively, the results indicate that cancer cell-intrinsic TGF-β signaling was demonstrated by multiplatform and high-resolution analyses in GC.

**Fig. 8:**
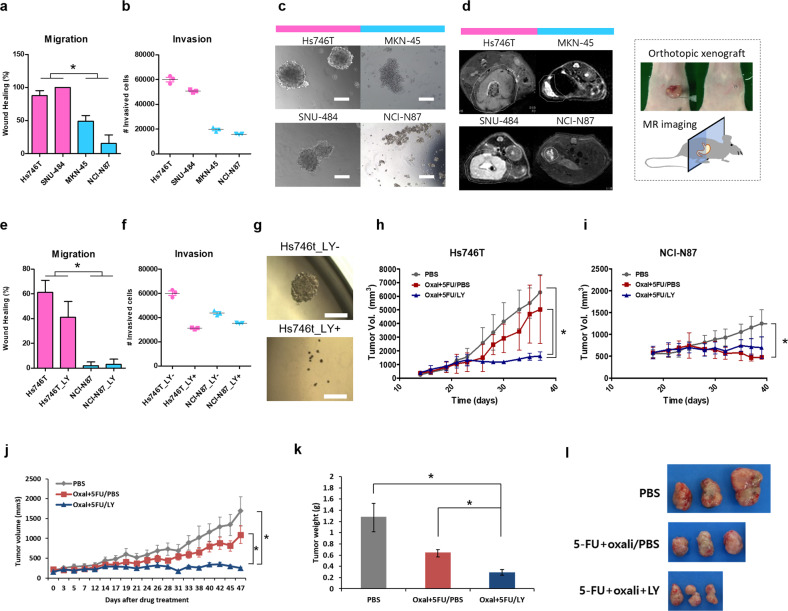
TGF-β inhibitor for chemoresistant GC preclinical models. **a**–**d** Phenotypes of the high (magenta) and low (sky blue) pink score GC lines were compared by **a** in vitro scratch wound-healing assay (*n* = 8) (*P* < 0.05), **b** invasion assay (*n* = 3), **c** in vitro tumor sphere formation assay (scale bars, 100 µm), and **d** in vivo orthotopic tumorigenesis. The diffuse growth of Hs746T and SNU-484 tumors and confinement of MKN-45 and NCI-N87 tumors are bordered by white dotted lines in the MRI images (axial section). Photographic illustration in the black dotted box depicts the orthotopic model construction. **e**–**i** Suppression of the mesenchymal behavior of Hs746T cells by treatment with the TGF-beta inhibitor (LY2157299 (LY)) was observed in the **e** in vitro scratch wound-healing assay (*n* = 20) (*P* < 0.05), **f** invasion assay (*n* = 3), and **g** in vitro tumor sphere formation assay. **h**, **i** In vivo drug-resistance assay measuring the tumor growth of **h** Hs746t tumors and **i** NCI-N87 tumors in a mouse xenograft model (*n* = 8) under the coadministration of a TGF-beta inhibitor during combination drug therapy (oxaliplatin and fluorouracil) (*P* < 0.05). **j**–**l** A high-risk, chemoresistant GC PDX model (GA077) treated with a TGF-beta inhibitor during combination drug therapy (*P* < 0.05). **j** PDX-bearing mice (*n* = 6 or greater) were treated with the indicated drug regimens. **k** Ex vivo tumor weights and **l** representative images of excised tumors.

Finally, we assessed the ability of a TGF-β inhibitor to attenuate mesenchymal/stem-like phenotypes in preclinical GC models. First, we selected Hs746T and SNU484 cell lines *versus* NCI-N87 and MKN-45 cell lines as the experimental models based on pink scores (GSE146361) (Supplementary Fig. 11). Accordingly, Hs746T and SNU484 cells demonstrated greater migration, invasion, and spheroid formation abilities than NCI-N87 and MKN-45 cells in vitro (Fig. 8a–c). Furthermore, magnetic resonance images of orthotopic models revealed that Hs746T and SNU484 cells diffused along the wall of the stomach over the course of tumor growth, whereas NCI-N87 and MKN-45 cells formed confined tumors in vivo (Fig. 8d). Treatment with galunisertib (LY2157299), a TGF-β inhibitor, delayed the migration, invasion, and spheroid formation of Hs746T cells in vitro without eliciting significant cytotoxicity (Fig. 8e–g). Disruption of TGF-β signaling in diffuse-type gastric carcinoma cell lines reduced pink module gene expression (GSE12336, Supplementary Fig. 12a). Next, we coadministered galunisertib and an anticancer drug combination (oxaliplatin+5-FU) to the xenograft mouse model established using Hs746T cells (*n* = 8). Although oxaliplatin + 5-FU treatment was only marginally effective against tumor growth in the Hs746T model, coadministration with galunisertib significantly attenuated the drug resistance and reduced the volume of Hs746T tumors in vivo (Fig. 8h). Conversely, the anticancer drug combination alone reduced tumor growth in NCI-N87 tumors without the aid of galunisertib (Fig. 8i). Moreover, we validated the efficacy of galunisertib in a GC PDX model previously described as the high-risk type^19^ and the EMT subtype^52^ (Fig. 8j–l). Treatment with galunisertib reduced the expression of genes in the pink module in a metastatic intestinal tumor model classified as CMS4 (GSE103562, Supplementary Fig. 12b). Taken together, pharmacological inhibition of TGF-β signaling may attenuate invasion, metastasis, and drug resistance in subtypes of GC with a dismal prognosis.

## Discussion

Gene expression profile-based classification has been widely accepted as a tool providing the groundwork for the development of precision medicine^7^. Molecular subtypes are relevant to cancer biology as well as clinical outcomes, so subtype classification facilitates the discovery of potential therapeutic targets. Moreover, the remarkable influence of nontumoral factors within the TME adds a layer of complexity for classification. The present consensus for the molecular classification of GC involves stroma-rich tumors and leucocyte-infiltrated tumors, which correspond to the MSC and INF subtypes in our study, respectively. The MSC subtype is consistent with diffuse, mesenchymal, GS, and EMT subtypes described in other classification systems. The INF subtype mainly overlaps with the EBV and MSI subtypes. Our classification system subdivided the CIN subtype, remaining TCGA subtype, into the GST, INT, and INT/S subtypes. The GST subtype showed high expression of the gastric gene module, including metabolic genes, and it was highly associated with two types of preneoplastic metaplasia (SPEM and IM) in GC progression^30^. The INT and INT/S subtypes exhibited high expression of intestine-specific genes and proliferative genes, but their prognoses strikingly varied across cohorts. In this study, we further conducted a deeper investigation focusing on the dismal prognoses of the MSC and INT/S subtypes.

We initially questioned whether the TME composition of the INT/S subtype was similar to that of the MSC subtype, but it was more similar to that of the INT subtype—with high tumor purity and small stromal component. In addition, neither tumor purity nor stromal score could significantly differentiate clinical outcome in our study. Therefore, we performed unsupervised network analyses to identify a common biological trait among the MSC and INT/S subtypes. First, WGCNA clustering detected a common “pink” gene module, which consisted of mesenchymal genes and was associated with the response to adjuvant treatments. Next, Ingenuity Pathway Analysis identified “TGFB1” as the upstream molecule that regulates gene expression of both dismal prognostic subtypes. This was substantiated by Connectivity Map database analysis, which revealed that the TGF-β inhibitors decreased the expression of the pink module genes to a greater degree than other treatments. The association of the pink module with TGF-β-induced EMT was further demonstrated by analyses of previously reported in vitro^47^ and in silico^48^ models. This is also in agreement with previous studies reporting activation of SMAD2/3, the canonical signaling molecule of the TGF-β pathway, in mesenchymal/EMT subtype GC^6,53^. Moreover, core TGF-β genes segregated poor prognostic and chemorefractory patients among the CIN/MSS subgroup. We validated the dependency of intractable subtype GC on TGF-β signaling in various preclinical experiments, including an experiment involving combination treatment in a PDX model. Our findings support that our GC classification system, which is based on a four gene-based commercial assay^19^, merits evaluation as a companion diagnostic test for subtyping and TGF-β inhibitor treatment guidance. Additionally, we anticipate results from ongoing clinical trials of the next-generation TGF-β inhibitor vactosertib^54^.

In this study, we also provided evidence for the presence of a partial EMT state and intrinsic expression of TGF-β signaling in GC cells. Above all, high-resolution spatial transcriptomics analysis showed differential expression and coexpression of epithelial and mesenchymal genes within adjacent lesions of a single primary GC. In addition, we identified the protein expression of SFRP4, a pink module gene with high compatibility between platforms and sample types^19^, in epithelial GC cells. Then, human array analysis of primary GC and derived preclinical models—with mouse stroma or without stromal content—showed that TGF-β pathway genes were consistently expressed with a very high correlation (R > 0.9). GSEA of scRNA-seq data also demonstrated activation of TGF-β signaling in cancer cells at a level similar to that in CAFs. These results are in agreement with those of recent studies: a scRNA-seq study showed that GC epithelial cells could be divided into two types based on EMT gene expression^55^, and a PDX study showed that cell-to-cell or extracellular matrix interactions were enriched in both GC cells and the TME^56^. Furthermore, *TGFB1* was expressed at higher levels in both primary GC deep lesions and metastatic lymph nodes than in primary GC superficial lesions, suggesting that this phenotype is cancer cell-related rather than tumor content-related^57^. Finally, scRNA-seq of superficial and deep lesions from diffuse-type GC identified that EMT and the TGF-β pathway were upregulated in malignant cells within deep lesions^58^.

Certain limitations should be noted in this study. First, the majority of analyses were performed using Asian patient cohorts. Second, downstream analysis was dependent on previously published data, although subtypes and gene modules were originally defined based on data-driven results. Third, survival analyses were performed using retrospective cohort data. Nonetheless, we believe our framework for therapeutic target discovery could provide useful information to facilitate the development of new treatment strategies. Most importantly, treatment with a TGF-β inhibitor should be considered for the selective treatment of patients with refractory subtype GC.

## Availability of data and material

The genomic data used in this study are available from the NCBI GEO with accession numbers described in each part. Spatial transcriptomic data will be shared upon appropriate request to the corresponding author.

## Supplementary information

Supplementary Figures, Tables, Results, and Discussion

Dataset 1-3
