# Author Correction: Clinical molecular subtyping reveals intrinsic mesenchymal reprogramming in gastric cancer cells

**DOI:** 10.1038/s12276-023-01037-6

**Published:** 2023-06-16

**Authors:** Eunji Jang, Min-Kyue Shin, Hyunki Kim, Joo Yeon Lim, Jae Eun Lee, Jungmin Park, Jungeun Kim, Hyeseon Kim, Youngmin Shin, Hye-Young Son, Yoon Young Choi, Woo Jin Hyung, Sung Hoon Noh, Jin-Suck Suh, Ji-Yong Sung, Yong-Min Huh, Jae-Ho Cheong

**Affiliations:** 1 MediBio-Informatics Research Center, Novomics Co., Ltd., Seoul, Republic of Korea; 2 College of Medicine, Yonsei University, Seoul, Republic of Korea; 3 Samsung Advanced Institute of Health Science and Technology, Sungkyunkwan University, Seoul, Republic of Korea; 4 Department of Pathology, Yonsei University, Seoul, Republic of Korea; 5 Department of Surgery, Yonsei University, Seoul, Republic of Korea; 6 Department of Radiology, Yonsei University, Seoul, Republic of Korea; 7 Department of Laboratory Medicine, Yonsei University College of Medicine, Seoul, Korea; 8 Department of Biomedical Systems Informatics, Yonsei University, Seoul, Republic of Korea; 9 YUHS-KRIBB Medical Convergence Research Institute, Seoul, Republic of Korea; 10 Department of Biochemistry & Molecular Biology, College of Medicine, Yonsei University, Seoul, Republic of Korea; 11 Brain Korea 21 Project for Medical Science, Yonsei University College of Medicine, Seoul, Republic of Korea

**Keywords:** Gastric cancer, Cancer genomics, Tumour heterogeneity, Cancer microenvironment, Classification and taxonomy

Correction to: Experimental & Molecular Medicine 10.1038/s12276-023-00989-z, published online 01 May 2023

In this article, the affiliation details for Min-Kyue Shin were incorrectly given as ‘Department of Digital Health, Samsung Advanced Institute of Health Science and Technology, Seoul, Republic of Korea’ but should have been ‘Samsung Advanced Institute of Health Science and Technology, Sungkyunkwan University, Seoul, Republic of Korea’.

The original article has been corrected.

