# Supplementary material for: Clinical molecular subtyping reveals intrinsic mesenchymal reprogramming in gastric cancer cells
Source: Exp Mol Med. 2023 May 1;55(5):974–86. doi: 10.1038/s12276-023-00989-z (PMC10238377; doi:10.1038/s12276-023-00989-z)
Supplement: Supplementary file 1 — Supplementary Figures, Tables, Results, and Discussion [file 12276_2023_989_MOESM1_ESM.pdf]

Supplementary Fig. 1 Consensus NMF clustering. Consensus maps and cophenetic coefficients with cluster numbers (k) 2 to 7 and the following variance cut-offs: (a) SD = 0.8, (b) SD = 0.9, and (c) SD = 1.0. (d) Sample assignment with silhouette width.

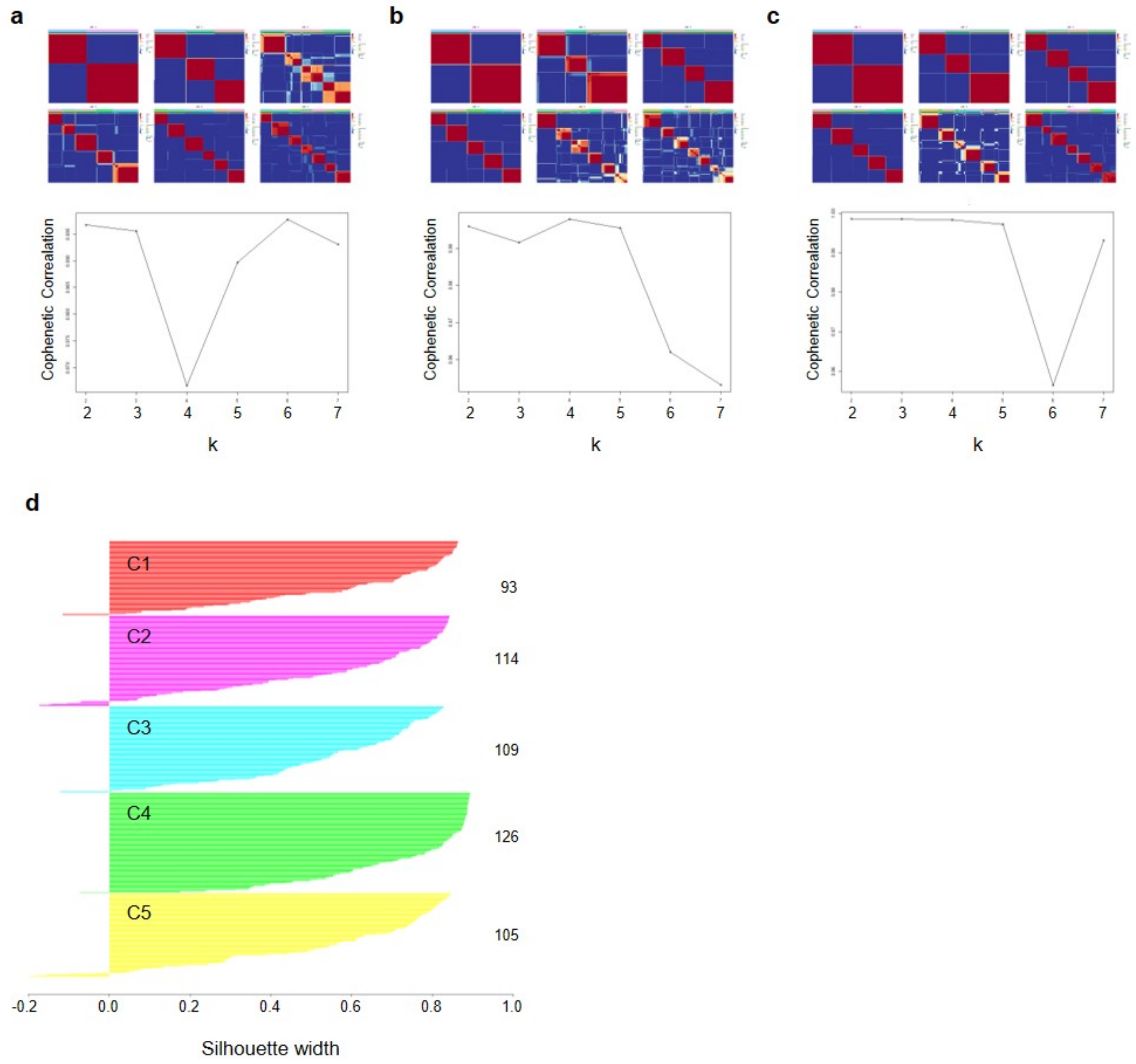

Supplementary Fig. 2 Module eigengene network for (a) all modules detected in YCC cohort and (b) the conserved modules, immune (red) and mesenchymal (blue) modules.

a

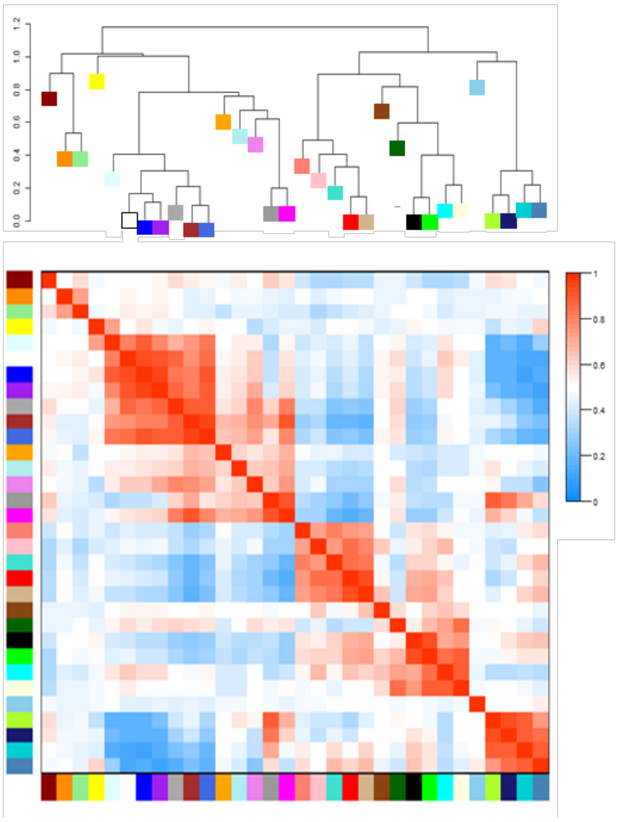

b

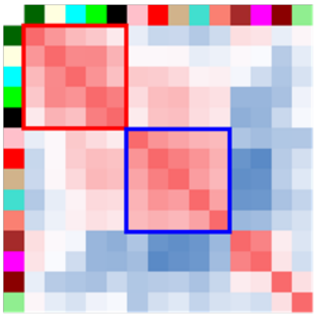

Supplementary Fig. 3 (a) Reverse Phase Protein Array (RPPA) expression of EMT associated proteins in STAD cohort (n = 305). Samples are sorted by EMT module enrichment score from left (low) to right (high). (b) Spearman correlation analysis between EMT module enrichment score and RPPA expression level.

a

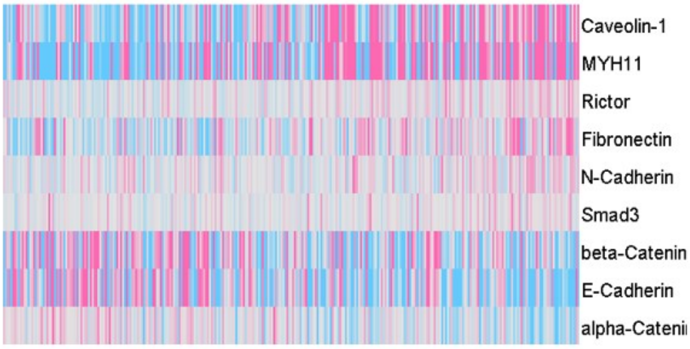

b

SYMBOL	P-value	correlation coefficient
CAV1	1.50E-10	0.357943
MYH11	4.73E-09	0.328751
RICTOR	9.22E-09	0.322739
FN1	8.31E-09	0.321898
CDH2	0.003325	0.167523
SMAD3	0.003441	0.166921
CTNNB1	0.002116	-0.17528
CDH1	1.40E-07	-0.29683
CTNNA1	1.46E-12	-0.39222

Supplementary Fig. 4 H&E-stained slide images of GC samples analysed with Visium platform.

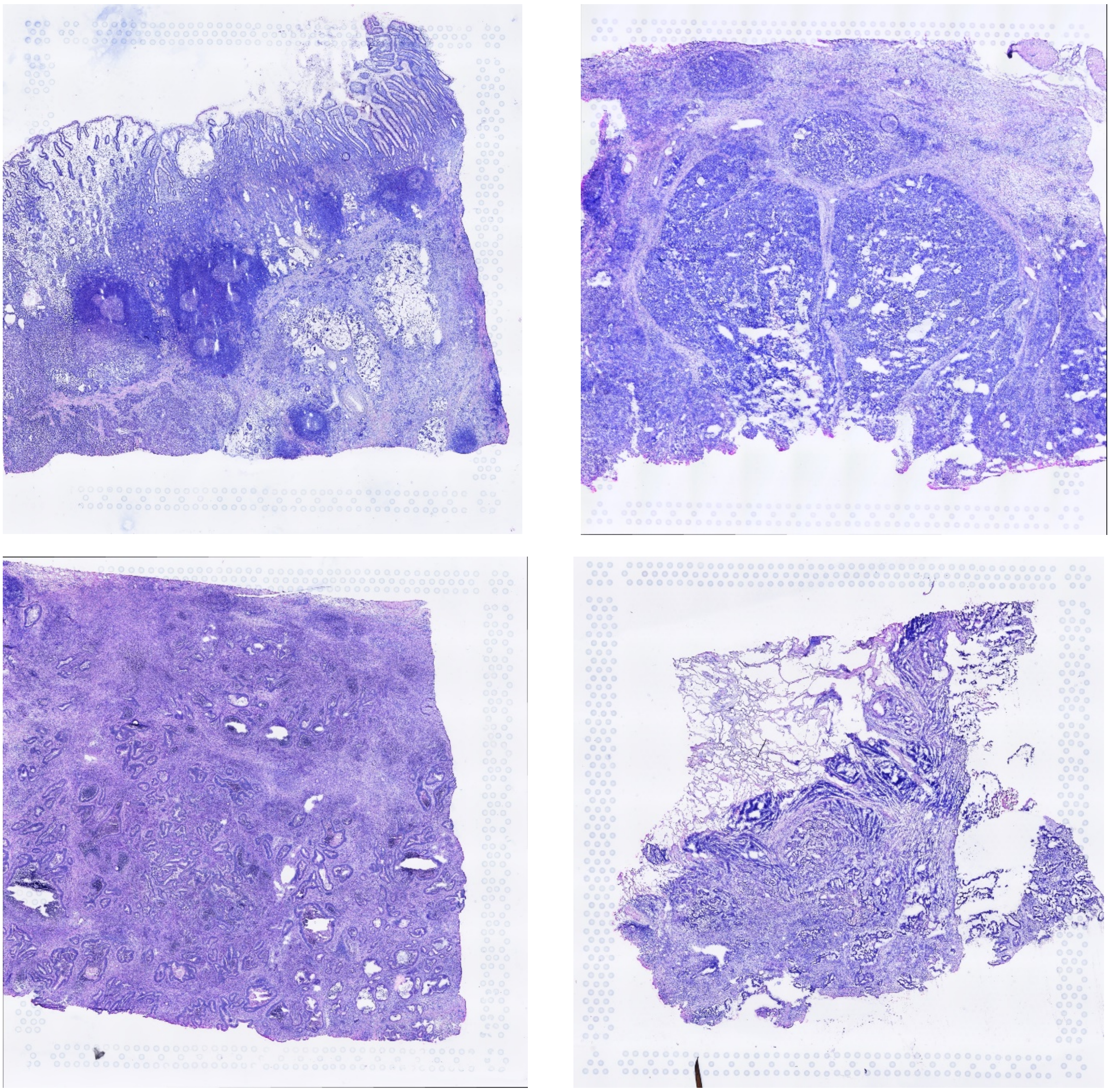

Supplementary Fig. 5 Immunostaining for SFRP4 and cytokeratin (CK) in a GC sample.

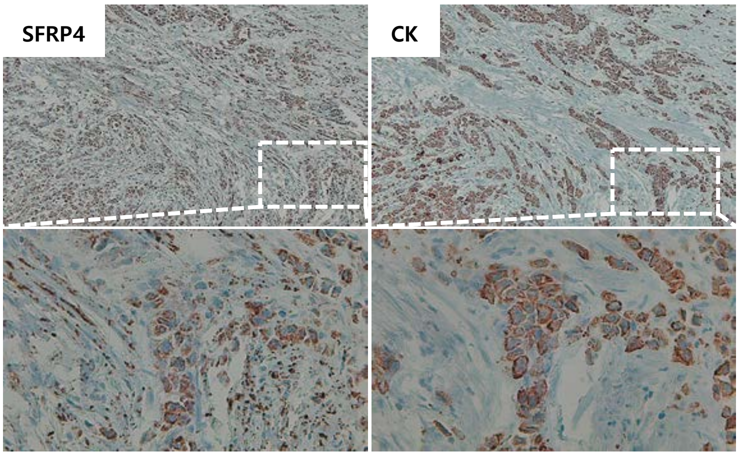

Supplementary Fig. 6 Pink module gene expression in gastric stem cells. (a-b) Pink module gene expression in (a) paired samples of AQP5+ and AQP5- populations collected from healthy human gastric pylori (GSE133036) and (b) human induced multipotent endodermal progenitors (hiMEPs) derived from gastric epithelial cells (GECs) (GSE58557).

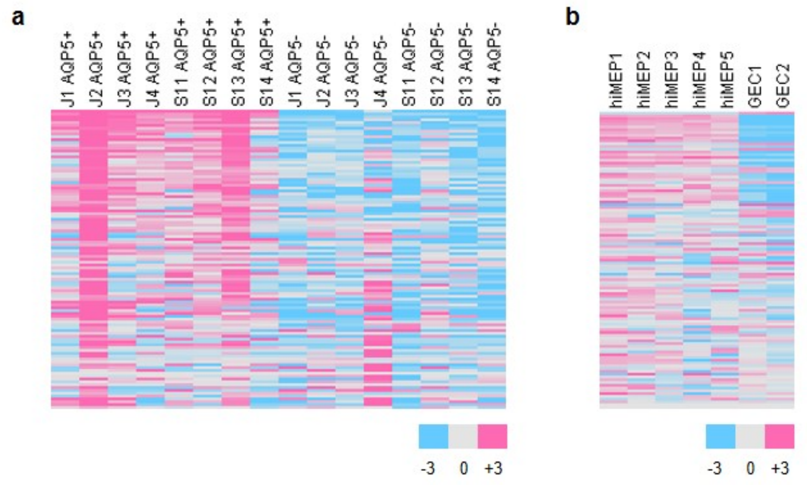

Supplementary Fig. 7 Pink module gene expression in breast tumor derived lung metastasis models. (a) Raw count sums of pink module genes in epithelial cells within metastatic niche (*n* =45) or distal parenchyma (*n* = 142) obtained from scRNA-seq data (GSE131508). (b) z-score means of pink module genes within dormant and proliferative cell model of metastasis (GSE120628). (*p* value calculated by the Wilcoxon test; center line, median; box limits, upper and lower quartiles; whiskers, 1.5× interquartile range within maximum/minimum values; points, outliers).

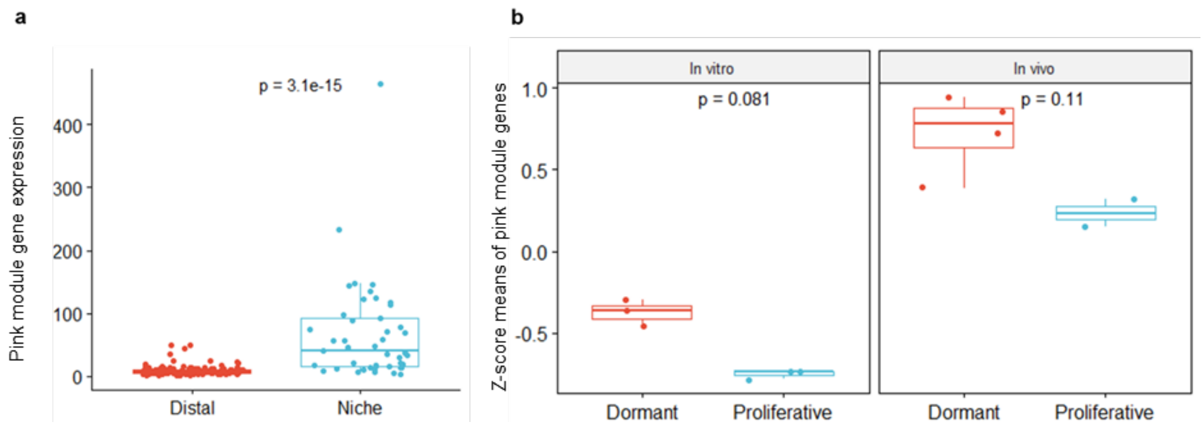

Supplementary Fig. 8 (a-b) Disease-free-survival among AJCC stage II-III patients (*n* = 193) who received adjuvant chemotherapy ± radiotherapy (CRT) and those who did not (GSE62254). Patients are divided by applying median cut-off to the pink module enrichment score and the xCell stroma score. (c) Subset analysis of GSE62254 patients with available estimated tumor purity from ASCAT analysis (*n* = 114). Patients are divided by applying median cut-off to the aberrant cell fraction. (d-e) Recurrence-free-survival among AJCC stage II-III patients (*n* = 178) who received standard adjuvant chemotherapy (CTX) and those who did not (pooled from GSE13861, GSE26942, and GSE147163). Patients are divided by applying median cut-off to the pink module enrichment score and the xCell stroma score for each cohort.

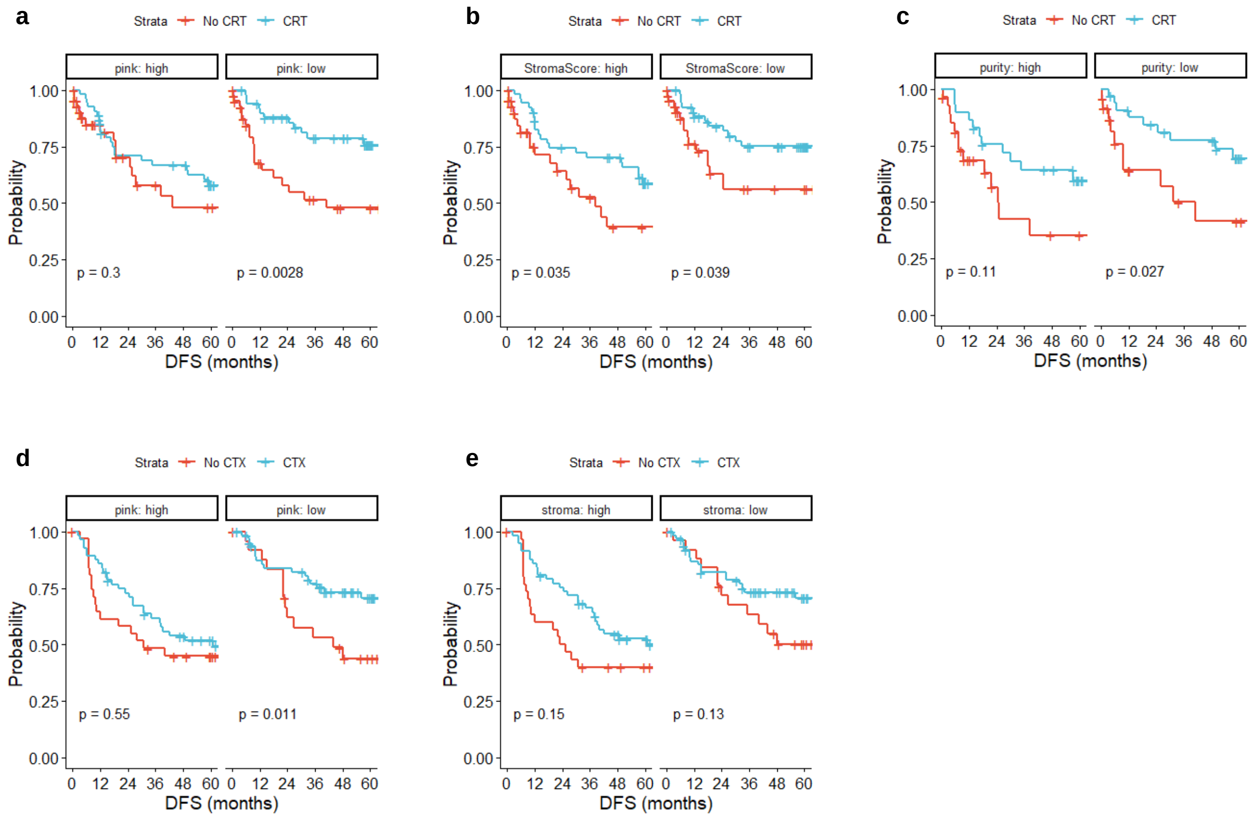

Supplementary Fig. 9 (a) Mean-centered log2(FPKM) values for pink module genes and response to 5-FU in Crownbio HuBase GC PDX models (n = 21). (b) Average expression level of pink module genes compared by response to 5-FU treatment (center line, median; box limits, upper and lower quartiles; whiskers, 1.5× interquartile range). (c-d) Overall survival among cohorts treated with (c) INT-0116 regimen adjuvant chemoradiotherapy (GSE26253) or (d) cisplatin and fluorouracil combination palliative chemotherapy (GSE14209).

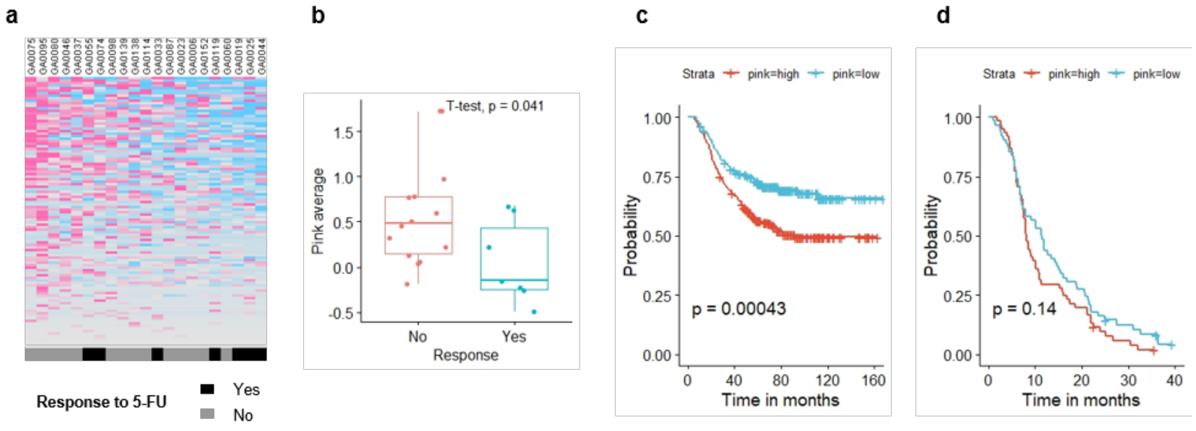

Supplementary Fig. 10 (a) Disease-free interval among TCGA CIN subtype patients (n = 122) stratified by single-sample gene set enrichment analysis (ssGSEA) of core TGF-β signalling genes. Median cut-off was applied. (b) Disease-free-survival among AJCC stage II-III patients with ACRG MSS/epithelial subtype (n = 123) who received adjuvant chemotherapy ± radiotherapy (CRT=1) and those who did not (CRT=0) (GSE62254). Patients are divided into low and high enrichment of core TGF-β signalling genes by applying median cut-off to the ssGSEA.

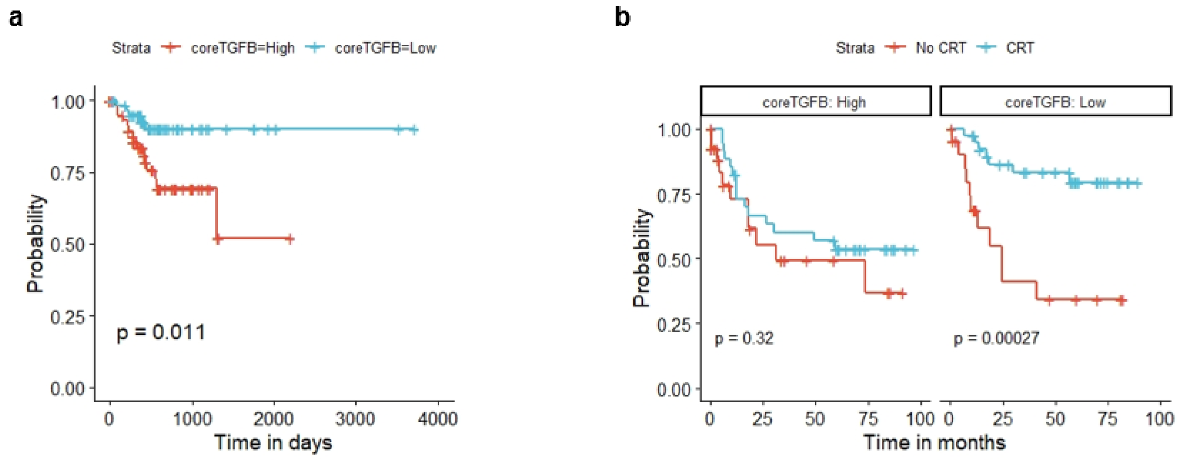

Supplementary Fig. 11 Pink scores of GC cell lines (GSE146361) calculated by single-sample gene set enrichment analysis.

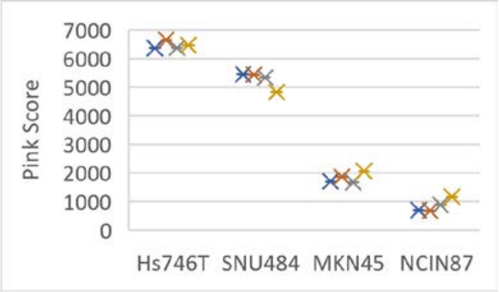

Supplementary Fig. 12 Change in the mesenchymal module gene expressions in (a) the diffuse-type gastric carcinoma cell lines (GSE12336, a dominant-negative TGF-β type II receptor (dnTβRII) or green fluorescent protein (GFP)) and (b) the metastatic intestinal tumor model displaying the CMS4 phenotype after treatment with Galunisertib (GSE103562, control and LY2157299 (Galunisertib)).

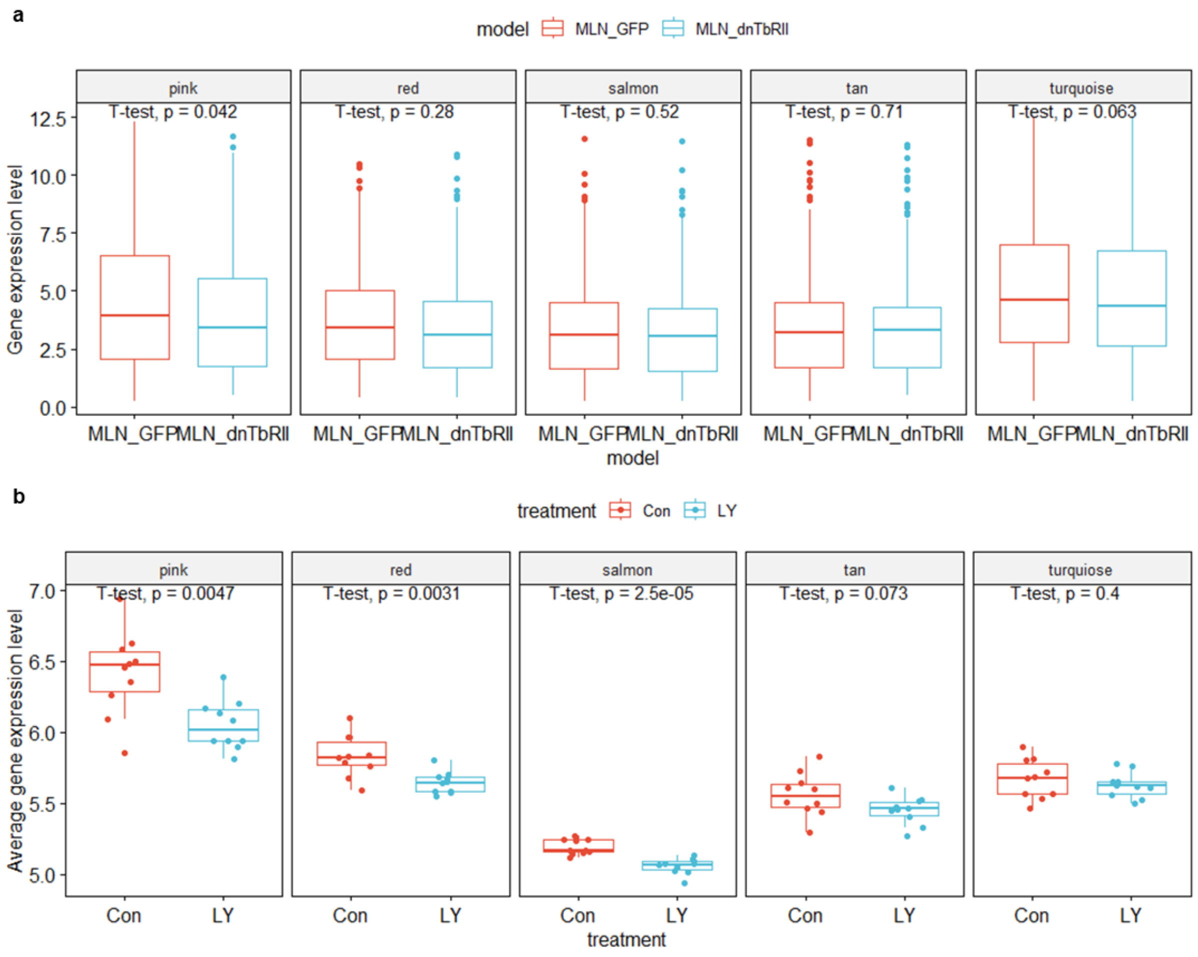

Supplementary Table 1 GSEA analysis of NMF consensus clustering in Training set I. Conditions for GSEA were set as one-others, pair-wise, or dummy comparison. The Molecular Signatures Database (MSigDB) and previous studies[1,2] were used for reference gene sets.

Comparison type	Subject1	Subject2	Gene set	Size	ES	NES	NOM p-val	FDR q-val
one-others	INF	others	Inflammatory response	142	0.636	2.30	0	0
			Interferon gamma response	157	0.771	2.47	0	0
			Interferon alpha response	79	0.809	2.38	0	0
	MSC	others	Myogenesis	133	0.630	2.13	0	6.E-03
			Epithelial mesenchymal transition	169	0.676	2.07	0	6.E-03
	GST	others	Digestion	28	0.730	2.28	0	0
			Spasmolytic polypeptide-expressing metaplasia	23	0.868	2.20	0	0
			Intestinal metaplasia	22	0.860	2.15	0	0
pair-wise	INT/S	MSC	G2M checkpoint	148	0.649	2.11	0	6.E-03
			E2F targets	157	0.709	2.10	0	3.E-03
	INT	MSC	G2M checkpoint	148	0.624	2.01	2.E-03	1.E-02
			E2F targets	157	0.659	1.93	2.E-03	2.E-02
	INT/S	INT	Epithelial mesenchymal transition	169	0.729	2.20	2.E-03	6.E-03
			Wnt signaling pathway	20	0.617	1.78	1.E-02	6.E-02
group-group	INT-INT/S-INF	GST-MSC	G2M checkpoint	148	0.714	2.31	0	0
			E2F targets	157	0.759	2.23	0	3.E-04

Abbreviations: ES, enrichment score; NES, normalized ES; NOM, nominal; FDR, false discovery rate; INF, inflammatory; INT, intestinal; GST, gastric; INT/S, INT with stem-like feature; MSC, stem-like.

Supplementary Table 2 Clinicopathological analysis of the five gastric cancer subtypes.

		GST	INF	MSC	INT/S	INT	p_{sub}	p
**Subtypes**		105	93	126	114	109	0.2533	0.2533
		19.2%	17.0%	23.0%	20.8%	19.9%		
age	> 60	51	53	53	71	75	0.07621	0.0002812
		16.8%	17.5%	17.5%	23.4%	24.8%		
	< 60	54	40	73	43	34	0.0007	
		22.1%	16.4%	29.9%	17.6%	13.9%		
sex	Male	69	69	78	83	70	0.6979	0.1477
		18.7%	18.7%	21.1%	22.5%	19.0%		
	Female	35	23	48	29	38	0.03531	
		20.2%	13.3%	27.7%	16.8%	22.0%		
location	Antrum	62	44	62	78	64	0.05143	-
		20.0%	14.2%	20.0%	25.2%	20.6%		
	Body Fundus	38	33	49	28	36	0.1587	
		20.7%	17.9%	26.6%	15.2%	19.6%		
	Cardia, GEJ	3	15	10	6	7	0.03882	
		7.3%	36.6%	24.4%	14.6%	17.1%		
	Whole	1	0	5	0	1	-	
		14.3%	0.0%	71.4%	0.0%	14.3%		
pT stage	T1&T2	15	10	4	16	16	0.06318	0.006754
		24.6%	16.4%	6.6%	26.2%	26.2%		
	T3	20	24	20	31	26	0.4773	
		16.5%	19.8%	16.5%	25.6%	21.5%		
	T4	69	58	102	65	66	0.002387	
		19.2%	16.1%	28.3%	18.1%	18.3%		
pN stage	N0	19	18	22	20	25	0.83	0.5142
		18.3%	17.3%	21.2%	19.2%	24.0%		
	N1	50	46	48	42	47	0.9443	
		21.5%	19.7%	20.6%	18.0%	20.2%		
	N2	25	22	45	40	30	0.0182	
		15.4%	13.6%	27.8%	24.7%	18.5%		
	N3	10	6	11	10	6	0.6096	
		23.3%	14.0%	25.6%	23.3%	14.0%		
AJCC stage	IA&IB	7	9	4	11	17	0.04186	0.1647

		14.6%	18.8%	8.3%	22.9%	35.4%		
	II	33	27	31	31	28	0.9384	
		22.0%	18.0%	20.7%	20.7%	18.7%		
	IIIA	35	32	38	34	32	0.9482	
		20.5%	18.7%	22.2%	19.9%	18.7%		
	IIIB	17	15	37	21	22	0.009656	
		15.2%	13.4%	33.0%	18.8%	19.6%		
	IV	12	9	16	15	9	0.4766	
		19.7%	14.8%	26.2%	24.6%	14.8%		
WHO classification	WD&MD	31	25	18	62	50	3.06E-07	1.45E-14
		16.7%	13.4%	9.7%	33.3%	26.9%		
	PD	46	57	67	32	43	0.005284	
		18.8%	23.3%	27.3%	13.1%	17.6%		
	SRC	15	0	27	3	4	2.35E-10	
		30.6%	0.0%	55.1%	6.1%	8.2%		
	Others	12	10	14	15	11	0.8464	
		19.4%	16.1%	22.6%	24.2%	17.7%		
Lauren type	Intestinal	34	37	23	58	41	0.002096	2.39E-11
		17.6%	19.2%	11.9%	30.1%	21.2%		
	Mixed	7	1	7	5	10	0.1193	
		23.3%	3.3%	23.3%	16.7%	33.3%		
	Diffuse	48	24	71	17	25	5.84E-11	
		25.9%	13.0%	38.4%	9.2%	13.5%		
	Others	15	30	25	32	32	0.1025	
		11.2%	22.4%	18.7%	23.9%	23.9%		

Abbreviations: GST, gastric; INF, inflammatory; MSC, mesenchymal; INT, intestinal; INT/S, INT with stem-like feature; GEJ, gastro-oesophageal junction; pT, pathological assessment of the primary tumor; pN, pathological assessment of the regional lymph nodes; WD, well differentiated; MD, moderately differentiated; PD, poorly differentiated; SRC, signet ring cell. The value of p_{sub} is from each hypergeometric test in the subcategory.

Supplementary Table 3 Comparison of the five gastric cancer subtypes with other subtypes/classifications.

Cohort	Subtype	INF	INT	GST	INT/S	MSC	sum	p_{sup}
GSE15459	Invasive	4	0	3	0	44	51	< 2.2e-16
		7.8%	0.0%	5.9%	0.0%	86.3%		
	Metabolic	1	16	21	0	2	40	1.06e-09
		2.5%	40.0%	52.5%	0.0%	5.0%		
	Proliferative	7	21	11	31	0	70	1.45e-08
		10.0%	30.0%	15.7%	44.3%	0.0%		
TCGA	EBV	18	4	0	0	2	24	1.11e-09
		75.0%	16.7%	0.0%	0.0%	8.3%		
	MSI	20	29	4	1	3	57	4.92e-11
		35.1%	50.9%	7.0%	1.8%	5.3%		
	GS	2	1	11	4	36	54	2.64e-16
		3.7%	1.9%	20.4%	7.4%	66.7%		
	CIN	8	20	30	49	20	127	1.82e-07
		6.3%	15.7%	23.6%	38.6%	15.7%		
GSE62254	MSS/T53-	6	25	29	34	13	107	0.000048
		5.6%	23.4%	27.1%	31.8%	12.1%		
	MSS/T53+	15	22	26	14	2	79	0.000274
		19.0%	27.8%	32.9%	17.7%	2.5%		
	MSI	31	25	8	3	1	68	5.12e-11
		45.6%	36.8%	11.8%	4.4%	1.5%		
	EMT	4	0	1	4	37	46	< 2.2e-16
		8.7%	0.0%	2.2%	8.7%	80.4%		

Abbreviations: INF, inflammatory; INT, intestinal; GST, gastric; INT/S, INT with stem-like feature; MSC, mesenchymal; EBV, Epstein-Barr virus; MSI, microsatellite instable; GS, genomic stable; CIN, chromosomal instability; MSS, microsatellite stable; EMT, epithelial-mesenchymal transition. The value of p_{sub} is from each hypergeometric test in the subcategory.

Supplementary Table 4 Gene ontology (GO) enrichment analysis of the conserved module.

Module	Module #[a]	GO biological process[b]	REF#	Merge#	Fold Enrichment	P value
turquoise	635	cytoskeleton organization	837	70	2.75	4.44E-10
		actin filament-based process	454	49	3.55	4.94E-10
		muscle system process	281	49	5.73	3.77E-18
red	115	regulation of cell migration	668	21	5.79	7.02E-07
		extracellular matrix organization	307	23	13.8	9.89E-16
pink	107	cellular response to transforming growth factor beta stimulus	149	7	9.13	1.57E-05
		extracellular matrix organization	307	31	19.64	3.64E-27
		connective tissue development	200	14	13.62	2.42E-08
		collagen metabolic process	74	16	42.06	1.67E-17
tan	85	vasculature development	478	21	10.89	2.49E-12
		Angiogenesis	297	14	11.68	1.45E-07
salmon	83	vasculature development	478	13	6.9	0.000403
		tube morphogenesis	330	11	8.46	0.000636
light green	56	Digestion	135	8	22.03	2.71E-05
dark red	44	carbohydrate metabolic process	668	11	7.79	0.000859
brown	155	mitotic cell cycle	754	50	8.9	2.35E-29
		cell division	473	37	10.5	6.62E-23
magenta	107	regulation of cell cycle process	555	25	8.84	5.58E-13
		cell division	473	27	11.21	6.95E-17
green	141	lymphocyte activation	344	15	6.58	9.69E-05
		adaptive immune response	277	17	9.26	5.4E-08
		leukocyte cell-cell adhesion	256	16	9.43	1.79E-07
black	108	leukocyte activation	416	23	10.75	1.88E-13
		lymphocyte activation	344	23	13.01	3.14E-15
		B cell activation	135	14	20.17	1.3E-10
cyan	79	innate immune response	609	21	9.09	7.18E-11
		adaptive immune response	277	12	11.41	5.56E-06
light yellow	48	cytokine-mediated signaling pathway	456	14	15.21	1.4E-09
		leukocyte activation	416	12	14.3	2.07E-07
		lymphocyte activation	344	11	15.85	5.39E-07
		response to virus	248	10	19.98	4.81E-07
dark green	38	cytokine-mediated signaling pathway	456	18	21.62	2.4E-16
		response to virus	248	17	37.55	3.58E-19
		response to type I interferon	67	15	> 100	4.79E-24

[a] # is the number of genes in the corresponding set of genes.
[b] Gene ontology, http://geneontology.org/.

Supplementary Table 5 Spearman correlation analysis between growth-inhibitory activity (log-fold change values of median fluorescence intensities relative to DMSO) of TGF beta receptor inhibitors and the pink module enrichment score in 578 human cancer cell lines (PRISM Repurposing dataset, https://depmap.org/repurposing/).

Name	Target	Drug category	Phase	Correlation coefficient	P-value
LY364947	TGFBR1	targeted cancer	Preclinical	-0.1184	0.0059
LY2157299	TGFBR1	targeted cancer	Phase 2/Phase 3	-0.0881	0.0388
SB-431542	ACVR1C, TGFBR1	targeted cancer	Preclinical	-0.0841	0.0494
adaprev	IGF2R, M6PR	noncancer	Phase 3	-0.0694	0.1109
pirfenidone	FURIN, TNF	noncancer	Launched	-0.0602	0.1587
F351	TGFB1	noncancer	Phase 2	-0.0578	0.1834
SD-208	TGFBR1	noncancer	Preclinical	-0.0389	0.3674
LY2109761	TGFBR1, TGFBR2	targeted cancer	Preclinical	-0.0189	0.6590
SB-525334	TGFBR1	noncancer	Preclinical	-0.0155	0.7178
D-4476	CSNK1A1, CSNK1D, TGFB1	noncancer	Preclinical	-0.0122	0.7796
repsox	TGFBR1	noncancer	Preclinical	0.0162	0.7053

Supplementary Methods

Pre-processing of microarray data sets for pattern-mining analysis

Three microarray data sets: GSE84437, GSE13861, and GSE147163, were quantile normalised using Between-Array Normalization (quantile) in the "Linear Models for Microarray Data (limma)" package and transformed into a $\log_2$ base. From these data sets, we preferentially excluded invalid probes in the Illumina BeadArray system according to Barbosa-Morais et al.[3] Each data set was then filtered by i) the variance in gene expression (S.D. thresholds from 0.8 to 1.2 by 0.1) to exclude potential noise and ii) common genes, using three data sets at the same S.D. threshold. Although all 547 samples were procured from a single platform (Illumina HumanHT-12 v3.0 Expression BeadChip array), possible technical biases needed to be considered. Individual data sets were therefore batch corrected and merged using the Bayesian algorithm "Combatting Batch Effects When Combining Batches of Gene Expression Microarray Data" (ComBat)[4]. The merged data set (YCC) was median centred, then applied to NMF clustering.

GSE15459 and GSE62254 were normalized using the R "affy" package including Robust Multi-array Average normalization.

Cell sorting from GC specimens and RNA sequencing

Primary tumor tissues were rinsed with Dulbecco's phosphate-buffered saline (Welgene LB00-02) with 2% antibiotics (Welgene LS203-01) and minced with a sterile blade. After filtering through a 0.2-μm syringe, the minced tissue was incubated with α-MEM (Gibco A10490) and 150 U/mL Collagenase II (Thermo Fisher Scientific) for 24 h at 37°C in a humidified atmosphere with 5% CO_2. The incubated tissue was centrifuged for 5 min at 200 × g, then transferred to fresh media. The minced tissue was passed through consecutive 18G and 21G needles and the enzymatic reaction of Collagenase IV was stopped by adding α-MEM with 10% fetal bovine serum (FBS). The suspension was filtered sequentially through cell strainers of 100, 70, and 40 μm. The filtered cells were collected by centrifugation for 10 min at 400 × g. The pellet was dispersed in 1.5 mL FACS buffer (PBS 50 mL + FBS 200 μL) and centrifuged for 5 min at 400 × g. The collected cells were incubated with antibody for 30 min on ice as follows: 10 μL anti-EpCAM (R&D Systems, FAB9601F) per 10^6 cells in 100 μL buffer; 5 μL anti-CD45 (BD, 557748) per 10^6 cells in 100 μL buffer; 10 μL anti-CD31 (Miltenyi Biotec, 130-092-652) per 10^7 cells in 100 μL buffer; and 2.5 μg of FAP (R&D systems, MAB3715) per 10^6 cells in 100 μL buffer. After washing with Hank's balanced salt solution (Lonza) twice, cells were incubated with mouse IgG (H+L) PE as follows: 10 μL per 10^6 cells in 100 μL buffer for 30 min on ice. After washing away the unstained secondary antibody, cells were resuspended in 1 mL PBS, then sorted using a BD FACSARIA III (BD Biosciences).

RNA purity was determined by assaying 1 μL total RNA extract on a NanoDrop8000 spectrophotometer. Total RNA integrity was checked using an Agilent Technologies 2100 Bioanalyzer with an RNA Integrity Number (RIN) value > 7 and the percentage of RNA fragments > 200 nt fragment distribution value (DV200). Total RNA sequencing libraries were prepared according to the manufacturer's instructions (Illumina TruSeq RNA Access Library kit). Next, 100 ng total RNA was fragmented into small pieces using divalent cations under elevated temperature. cDNA was generated from the cleaved RNA fragments using random priming during first and second strand synthesis, and sequencing adapters were ligated to the resulting double-stranded cDNA fragments. The coding regions of the transcriptome were then captured from this library using sequence-specific probes to create the final library. The quality of the amplified libraries was verified by capillary electrophoresis (Bioanalyzer, Agilent). After qPCR using a SYBR Green PCR Master Mix (Applied Biosystems), we combined libraries that were index tagged in equimolar amounts. Cluster generation was performed in the flow cell on the cBot automated cluster generation system (Illumina). The flow cell was then loaded on a HiSeq 2500 sequencing system (Illumina) and sequencing was performed using 2 × 100 bp read lengths.

In vitro and *in vivo* experimental validation

Cell lines: The human gastric cancer cell lines SNU-1, SNU-5, SNU-16, SNU-216, SNU-484, SNU-520, SNU-601, SNU-620, SNU-638, SNU-668, SNU-719, MKN-1, MKN-28, MKN-45, MKN-74, KATOIII, NCI-N87, and Hs746T were purchased from the Korean Cell Line Bank (Seoul, Korea); and YCC-1, YCC-2, YCC-3, YCC-6, YCC-7, YCC-9, YCC-10, YCC-11, and YCC-16 were purchased from the Yonsei Cancer research Institute (Seoul, Korea). SNU-1, SNU-5, SNU-16, SNU-216, SNU-484, SNU-520, SNU-601, SNU-620, SNU-638, SNU-668, SNU-719, MKN-1, MKN-28, MKN-45, MKN-74, KATOIII, and NCI-N87 were grown in RPMI 1640 (Welgene, Daegu, Korea); Hs746T in Dulbecco's modified Eagle's medium (DMEM, Welgene); and YCC-1, YCC-2, YCC-3, YCC-6, YCC-7, YCC-9, YCC-10, YCC-11, and YCC-16 in Minimum essential medium (MEM, Welgene). All cells were cultured in complete media supplemented with 10% FBS (Gibco) and 1% antibiotic-antimycotic solution (including 10,000 units of penicillin, 10 mg streptomycin, and 25 μg amphotericin B per mL, Sigma-Aldrich) at 37°C in a humidified atmosphere containing 5% CO_2. All the cells were confirmed to be negative for mycoplasma using the e-Myco™ plus Mycoplasma PCR Detection Kit (iNtRON Biotechnology, Seongnam, Korea). All *in vitro* experiments were performed using cell lines with a low passage number (4 to 10 passaging cycles after recovery of cryopreserved cells).

Supplementary Results and Discussion

Unsupervised GC sample clustering: consensus NMF clustering

GC molecular subtypes were discovered by the unsupervised clustering of GC samples with a stochastic algorithm of NMF. NMF mathematically reduces the dimension of gene expression data to a few metagenes, which enables the analysis of samples in terms of the expression pattern of the metagene. As NMF works by an iterative approximation that depends on starting condition, multiple runs with different initializations are essential to achieve stability. This process is visualized with a consensus map and quantitatively evaluated by computing the cophenetic correlation coefficient (ρ_k) to help select the appropriate number of clusters (k), as shown in **Supplementary Fig. 1a–c.**

From the NMF results with *n* = 547 samples, we selected k = 5, which achieved consensus across the range of S.D. cut-offs, where all ρ_5 values were higher than 0.99. Although ρ_2 and ρ_3 also satisfied the consensus limit, k = 2 and 3 were preferentially ruled out. We assumed that such a low level of classifications could not embrace the heterogeneity of GC. For k = 4 and 6, blurry boundaries in their consensus plotting, depending on the *a priori* selection of genes, demonstrated that they were rather metastable. Finally, we chose k = 5 at the S.D. cut-off 0.9 (1603 genes), where the magnitude of ρ_5 begins to fall, as being consistent with the model selection methodology suggested by Brunet et al.[5]

The five GC subtypes were annotated based on on the collective expression of pre-defined gene sets relevant to GC biology and results from gene set enrichment analysis (GSEA) (**Supplementary Table 1**).

Identification of NMF-based GC subtypes and subtype classifiers

Before analysing the GC subtypes, we additionally filtered out genes that contribute less to discriminating GC clusters based on the significance analysis of microarrays (SAM)[6] and prediction analysis of microarrays (PAM)[7]. To this end, we excluded 15 sample outliers with negative silhouette width[8] in advance, which did not statistically belong to the assigned cluster (**Supplementary Fig. 1d**). This exclusion helped the subsequent SAM and PAM analyses to focus on subtype-specific characteristics. In general, SAM calculates *t*-statistics for each gene to determine genes that are significantly differentially expressed across subtypes. PAM also uses the *t*-statistics with the nearest shrunken centroid method to sort the genes that best characterize specific subtypes. From the NMF-clustered gene expression data of *n* = 532 samples with the outliers removed, SAM found 965 genes to be significantly differentially expressed across the five subtypes at the threshold (Δ_{SAM}) of 11.8 with false discovery rates (FDR) of zero. The subsequent PAM evaluated genes based on their contribution to subtype characterization by varying shrinkage (Δ_{PAM}). The results of SAM and PAM are listed in **Supplementary Data 1.**

Prior to the annotation of subtypes, we conducted gene set enrichment analysis (GSEA)[9] to determine whether an *a priori*-defined set of genes was significantly correlated with the GC subtypes derived by NMF (**Supplementary Table 1**). Considering the precondition of GSEA analysis that compares two expression profiles, we established various types of comparison groups: one-others, pairwise, and group-group comparisons. A subtype can be characterized by one or more molecular patterns of gene expression. If a molecular pattern of gene expression is specific to a single subtype, then this signature would be detected by one-others or pairwise comparison. Another molecular pattern of gene expression might be shared by a few subtypes, in which case a group-group comparison would discover such signatures across subtypes. For the analysis, we mainly used a hallmark gene set, KEGG gene sets, and GO gene sets from Molecular Signatures Database v5.1. Based on GSEA, we found several trends (described below as i–iv). In one-others comparison, (i) cluster 1 was significantly associated with the inflammatory response (FDR = 0), prompting us to annotate cluster 1 as an inflammatory (INF) subtype. (ii) Cluster 4 was associated with myogenesis (FDR = 5.66e-3), epithelial-mesenchymal transition (FDR = 5.71e-3), and angiogenesis (FDR = 5.42e-2). We thus annotated cluster 4 as a mesenchymal (MSC) subtype. (iii) Clusters 2 and 3, which were similar in their high expression of intestinal epithelial cell differentiation markers (CDH17 and CDX2), were commonly associated with the cell division cycle (G2/M check-point, FDR = 5.71e-3 and 0.012, respectively; E2F targets, FDR = 2.86e-3 and 0.017, respectively) compared to the MSC subtype. iv) Cluster 2, however, appeared to represent epithelial-mesenchymal transition (FDR = 1.97e-3) when compared to cluster 3. Thus, we annotated cluster 3 as the intestinal (INT) subtype and cluster 2 as the INT with stem-like feature (INT/S) subtype. v) Notably, the cell cycle-related characteristics were shared not only by the INT and INT/S subtypes, but also by the INF subtypes. In the group-group comparisons, the INT, INT/S, and INF subtypes were significantly associated with the G2/M check point (FDR = 0) compared to the rest of the clusters. vi) Cluster 5 was significantly associated with digestion (FDR = 0), as well as two metaplasias (spasmolytic polypeptide-expressing metaplasia, FDR = 0; intestinal metaplasia, FDR = 0). We thus annotated cluster 5 as a gastric subtype.

WGCNA analysis to discover GC molecular signatures

To support the annotation of NMF-derived subtypes by GSEA, we introduced weighted gene co-expression network analysis (WGCNA)[10] to translate subtype-specific characteristics into gene-wise network language. Whereas the S/PAM analyses characterize GC subtypes by filtering and ranking subtype-differentially expressed genes (DEGs), WGCNA describes the connectivity among genes. In high-dimensional gene expression profiles, WGCNA constructs a gene correlation network to find modules that consist of closely interconnected genes. By analysing modules in gene expression data in conjunction with external clinical information, we could identify the key gene(s) leading to the molecular signatures of GC.

In WGCNA, a co-expression network is constructed by measuring the pairwise relationships between genes across the data set and transforming them into a weighted adjacency matrix with

soft-thresholding. This weighted gene network is scale-free and mimics common networks in nature. As the adjacency matrix is constructed, the network distance measure, known as the topological overlap dissimilarity measure, is utilized to bring out average linkage hierarchical clustering, where we detect gene modules as branches of the resulting dendrogram[11]. Selecting a hybrid method with a split number of four resulted in the detection of 32 modules. The largest module (turquoise) included 635 genes, whereas the remaining modules ranged from 165 to 23 genes. Gene lists in each module are provided as **Supplementary Data 2.** To summarize each module, we calculated module eigengenes (MEs), defined as the first principal component of a given module. MEs can be used as a representative of the gene expression profiles in a module.

Prior to examining the association of WGCNA modules with the GC subtypes, we prioritized the GC modules by analysing whether a module can be consistently detected in the independent cohorts, Singapore, TCGA, and ACRG. Via a hypergeometric test between modules across the cohorts, 14 modules were prioritised to be conserved, including dark red, light green, magenta, brown, salmon, red, pink, turquoise, tan, dark green, black, green, cyan, and light yellow. For convenience, the colour of the detected modules in each cohort was synchronized with the colours of the GC modules identified in the Training set I when the majority of genes in the modules overlapped with corresponding modules in the training set. We analyzed the conserved modules using gene ontology enrichment analysis (**Supplementary Table 4**). The intermodular correlations of the conserved modules were shown in **Supplementary Fig. 2**. We characterized the conserved modules, and subsequently used them to define six GC molecular signatures (**Main Text**).

We analyzed the correlation of modules with the five GC subtypes and clinical information using MEs. The point-biserial correlation of MEs with subtype categories was shown in **Fig. 1h.** Briefly, two immune and mesenchymal modules were strongly associated with the INF and MSC subtypes, respectively, across four cohorts (point-biserial correlation $\rho > 0.5$, $P < 0.05$). The gastric module was exclusively correlated with the GST subtype in all cohorts (point-biserial correlation $\rho > 0.4$, $P < 0.05$). The INT and INT/S subtypes were moderately governed by multiple common modules, including the proliferative module. However, two subtypes were distinctive with regard to the intestinal and stem-like modules. The stem-like module in all cohorts was clearly specific to the INT/S subtype. Notably, the INT/S and MSC subtypes with bad prognoses were both positive for the pink module, associated with *Wnt* and *TGF* signaling-related genes.

To examine the relationship between GC modules and clinical information, we examined the correlation of modules with clinical information, including age, sex, tumor location, pTstage, pNstage, AJCC stage, WHO classification, Lauren type, and recurrence (**Supplementary Data 3**). The results were as follows: i) The mesenchymal modules were positively correlated with age under 60, pTstage 3, PD and SRC in the WHO classification, diffuse Lauren type, and recurrence. This trend was similar to the association of the MSC subtype with clinical information (**Supplementary Table 2**). ii) The proliferative module, however, showed a positive correlation with age above 60, pTstage 1&2, AJCC IA&IB, WD&MD in the WHO classification, and intestinal Lauren type. iii) The immune modules were slightly correlated with the cardiac esophagogastric mucosal junction in tumor location and with PD in the WHO classification.

Comparison of the five GC subtypes with published subtypes

We compared the similarities and differences of our classification with the GC subtypings reported by the Singapore research group (GSE15459)[12], TCGA (TCGA)[13], and Asian Cancer Research Group (ACRG) (GSE62254)[14]. The contingency table is summarized in **Supplementary Table 3**.

The Singapore research group used consensus hierarchical clustering with iterative feature selection and categorized GC into three subtypes: invasive (mesenchymal), proliferative, and metabolic. We made the following observations: i) The majority (86.3%) of samples in the invasive subtype was classified as our MSC subtype (hypergeometric test; P < 2.2e-16). Both subtypes shared the stem-like properties of high expression of EMT and mesenchymal genes and had histologically strong associations with Lauren diffuse type and poorly differentiated GC. ii) Samples in the metabolic subtype belonged to our GST and INT subtypes (52.5% and 40.0%, respectively). The distinctive activity of a pathway related to spasmolytic-polypeptide-expressing metaplasia (SPEM) was commonly observed in the metabolic and GST subtypes. iii) In the proliferative subtype, samples were characterized by the high expression of cell cycle pathways and 84.3% were distributed across our INF, INT, and INT/S subtypes. Owing to upregulated expression of cell cycle-related genes, seven of the twelve samples assigned to our INF subtype were of the proliferative subtype. Conversely, our MSC subtype specifically demonstrated low cell cycle-related gene expression.

TCGA established an integrative classification algorithm based on multiple analysis platforms and defined four subtypes: Epstein-Barr virus (EBV) positive, microsatellite instability (MSI), genome stability (GS), and chromosomal instability (CIN). Between the TCGA subtypes and ours, the following observations can be made: i) Highly distinctive frequencies were observed in the EBV and GS subpopulations, wherein 75.0% of EBV samples were classified as our INF subtype and 66.7% of GS samples were classified as our MSC subtype (hypergeometric test; P = 1.1e-9 and 2.6e-16, respectively). ii) MSI subtype samples were distributed between our INF and INT subtypes (35.1% and 50.9%, respectively; hypergeometric test; P = 4.9e-11). A total of 80% of our INF subtype samples consisted of the EBV and MSI subtypes. iii) The CIN subtype was spread over five subtypes, although 90% of our INT/S subtype samples were CIN. TCGA classified the CIN subtype based on the existence of any chromosomal alteration, which could not specify functional varieties in our subtype but reflected the etiological bases.

The ACRG classification identified four GC subtypes: EMT, MSI, MS stable with TP53 positive (MSS/TP53+), and MS stable with TP53 negative (MSS/TP53−), which were established by gene set-based principal component analysis (PCA) and TP53 activity. i) Most markedly, 80.4% of EMT subtype samples were classified as our MSC subtype (hypergeometric test; P < 2.2e-16). ii) The MSI subtypes overlapped partially owing to the INF and INT subtypes (45.6% and 36.8%, respectively, hypergeometric test; P = 5.1e-11). iii) Our INF subtype contained 11/18 EBV-positive samples (hypergeometric test; P = 4.038e-7),

whereas the ACRG subtype was not associated with EBV infection. iv) MSS/TP53− in non-EMT and non-MSI samples appeared more likely associated with INT/S and MSC subtypes than MSS/TP53+. In the MSS/TP53+ and INF subtypes, 8/15 were EBV-positive.

Taken together, the test cohorts exhibited two main trends: i) our MSC subtype was a consensus subtype across the three classification systems (Singapore research group invasive subtype, TCGA GS subtype, and ACRG EMT subtype), and ii) our INF subtype included most EBV-positive patients and a partial high-MSI group identified by TCGA and ACRG. Otherwise, our GST and INT subtypes were partially described by the Singapore research group. The INT/S subtype was putatively associated with structural chromosomal instability by TCGA. For the non-MSC and non-INF GC population, subtype matching tended to be pathologically ambiguous, likely because of the differences in classifier type, where TCGA and ACRG chose the degree of somatic copy-number aberrations and TP53 activity, respectively, as classifiers.

<u>**References for Supplementary Results and Discussion**</u>

1 Lee, H. J. *et al.* Gene expression profiling of metaplastic lineages identifies CDH17 as a prognostic marker in early stage gastric cancer. *Gastroenterology* **139**, 213-225.e213 (2010).

2 Weis, V. G. & Goldenring, J. R. Current understanding of SPEM and its standing in the preneoplastic process. *Gastric cancer* **12**, 189-197 (2009).

3 Barbosa-Morais, N. L. *et al.* A re-annotation pipeline for Illumina BeadArrays: improving the interpretation of gene expression data. *Nucleic Acids Res.* **38**, e17 (2010).

4 Chen, C. *et al.* Removing batch effects in analysis of expression microarray data: an evaluation of six batch adjustment methods. *PloS one* **6**, e17238 (2011).

5 Brunet, J. P., Tamayo, P., Golub, T. R. & Mesirov, J. P. Metagenes and molecular pattern discovery using matrix factorization. *Proc. Natl. Acad. Sci. U. S. A.* **101**, 4164-4169 (2004).

6 Tusher, V. G., Tibshirani, R. & Chu, G. Significance analysis of microarrays applied to the ionizing radiation response. *Proc. Natl. Acad. Sci. U. S. A.* **98**, 5116-5121 (2001).

7 Tibshirani, R., Hastie, T., Narasimhan, B. & Chu, G. Diagnosis of multiple cancer types by shrunken centroids of gene expression. *Proc. Natl. Acad. Sci. U. S. A.* **99**, 6567-6572 (2002).

8 Rousseeuw, P. J. Silhouettes - a Graphical Aid to the Interpretation and Validation of Cluster-Analysis. *J. Comput. Appl. Math.* **20**, 53-65 (1987).

9 Subramanian, A. *et al.* Gene set enrichment analysis: a knowledge-based approach for interpreting genome-wide expression profiles. *Proc. Natl. Acad. Sci. U. S. A.* **102**, 15545-15550 (2005).

10 Langfelder, P. & Horvath, S. WGCNA: an R package for weighted correlation network analysis. *BMC bioinformatics* **9**, 559 (2008).

11 Langfelder, P., Zhang, B. & Horvath, S. Defining clusters from a hierarchical cluster tree: the Dynamic Tree Cut package for R. *Bioinformatics* **24**, 719-720 (2008).

12 Lei, Z. *et al.* Identification of molecular subtypes of gastric cancer with different responses to PI3-kinase inhibitors and 5-fluorouracil. *Gastroenterology* **145**, 554-565 (2013).

13 Comprehensive molecular characterization of gastric adenocarcinoma. *Nature* **513**, 202-209 (2014).

14 Cristescu, R. *et al.* Molecular analysis of gastric cancer identifies subtypes associated with distinct clinical outcomes. *Nat. Med.* **21**, 449-456 (2015).
